# GC_3 _biology in corn, rice, sorghum and other grasses

**DOI:** 10.1186/1471-2164-11-308

**Published:** 2010-05-16

**Authors:** Tatiana V Tatarinova, Nickolai N Alexandrov, John B Bouck, Kenneth A Feldmann

**Affiliations:** 1 Department of Biomedical Engineering, Georgia Institute of Technology, Atlanta, Georgia, 30332, USA; 2 Division of Mathematics and Statistics, University of Glamorgan, Pontypridd, CF37 1DL, UK; 3 Ceres, Inc, 1535 Rancho Conejo Rd, Thousand Oaks, CA, 91320, USA; 4 School of Plant Sciences, University of Arizona, Tucson, AZ, 85721, USA

## Abstract

**Background:**

The third, or wobble, position in a codon provides a high degree of possible degeneracy and is an elegant fault-tolerance mechanism. Nucleotide biases between organisms at the wobble position have been documented and correlated with the abundances of the complementary tRNAs. We and others have noticed a bias for cytosine and guanine at the third position in a subset of transcripts within a single organism. The bias is present in some plant species and warm-blooded vertebrates but not in all plants, or in invertebrates or cold-blooded vertebrates.

**Results:**

Here we demonstrate that in certain organisms the amount of GC at the wobble position (GC_3_) can be used to distinguish two classes of genes. We highlight the following features of genes with high GC_3 _content: they (1) provide more targets for methylation, (2) exhibit more variable expression, (3) more frequently possess upstream TATA boxes, (4) are predominant in certain classes of genes (e.g., stress responsive genes) and (5) have a GC_3 _content that increases from 5'to 3'. These observations led us to formulate a hypothesis to explain GC_3 _bimodality in grasses.

**Conclusions:**

Our findings suggest that high levels of GC_3 _typify a class of genes whose expression is regulated through DNA methylation or are a legacy of accelerated evolution through gene conversion. We discuss the three most probable explanations for GC_3 _bimodality: biased gene conversion, transcriptional and translational advantage and gene methylation.

## Background

**Figure 1:**
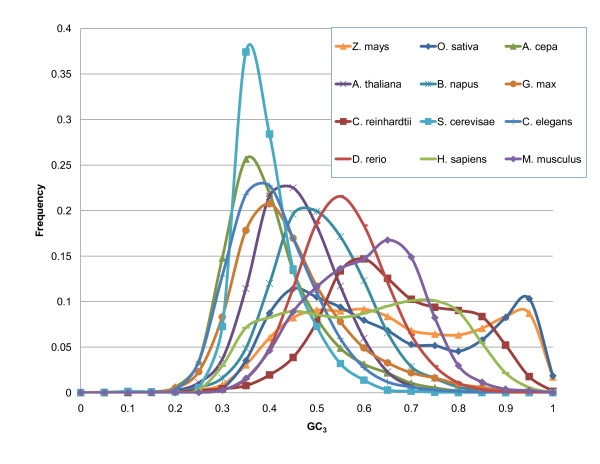
**GC_3 _distributions**. Distribution of GC_3 _is bimodal for Z. *mays, O. sativa, C. reinhardtii *and *H. sapiens*. Other organisms are either AT-rich, *A. cepa, A. thaliana, G. max, S. cerevisiae *and *C. elegans*, or located between the two groups, *B. napus, D. rerio *and *M. musculus*.

Examination of the nucleotide content of various transcriptomes has revealed classes of genes distinguished by their G and C content [1]. In particular, the wobble position of coding sequences, which is relatively independent of the coding potential of the protein, serves as a marker for GC richness. The frequency of G+C nucleotides at the 3^rd ^position is referred to as GC_3_. In several earlier studies [2-5], two types of organisms were identified on the basis of their GC_3 _distributions: unimodal and bimodal. Warm-blooded animals and several plant families (*Poaceae*, *Musaceae *and *Zingiberaceae*) demonstrate a clear bimodal distribution of GC_3 _while cold-blooded animals and other plants (including dicots) show a predominantly unimodal distribution (Figure 1).

Since bimodality has been detected in only some plant families, we suggest that this feature has developed independently in warm-blooded animals and in certain members of the commelinids clade. The GC_3 _bias could possibly be explained as a consequence of some larger genomic bias. For example, over three decades ago, Macaya *et al*. [6] observed that some genomes contain isochores, megabase-long regions with either high or low GC contents. Isochores have been reported in warm-blooded vertebrates and in some reptiles [7-9]. Compositionally homogenous DNA regions of at least 50-100 kb have been found in several dicot and monocot genomes (pea, sunflower, tobacco, barley, rice, maize, oat and wheat), supporting the existence of isochores in plants [10-12]. It is not yet known whether all eukaryotic genomes are characterised by an isochore structure [9].

Press and Robins [13] reported that high GC isochores contain a mixture of GC- and AT-rich genes, whereas high AT (low GC) isochores contain mostly AT-rich genes. Genes found within high and low GC isochores are functionally distinguishable by statistical analysis of their gene ontology categories [13]. The authors suggested that some genes require AT-richness, while others, contained within large coherent blocks, have a strong bias towards mutations to GC.

The neutral theory of evolution states that for a change to come about in the population as a whole, the new characteristic must be as good as or better than the old one. Under the assumption of neutrality, genes would acquire characteristics of the surrounding isochores. Therefore, noncritical elements such as synonymous bases in 3^rd ^codon positions and 5' and 3' UTRs should be GC-rich in high GC isochores. In fact, several groups have found a positive correlation between the GC_3 _levels of a gene and of its surrounding genomic area [2,13,14]. Mouchiroud *et al*. [15] found an 8-fold enrichment for high-GC_3 _genes within the top 3% of the GC-richest isochores in humans. These observations support the neutrality assumption. Elhaik *et al*. [16], however, found little correlation between GC_3 _and isochores within a species and none between species. Furthermore, the correlation with generally GC-rich areas is only modest (R^2 ^= 0.43) [17], suggesting that a more complex explanation must be sought. Moreover, isochores have been reported in both GC_3 _unimodal and bimodal organisms and therefore cannot provide an exclusive explanation for GC_3 _bimodality.

Campbell and Gowri [1] described differences in codon usage in different plant genomes, algae and cyanobacteria, and showed that bimodality existed only in monocots. In a series of publications [10,11], GC_3 _levels were analyzed for five *Poaceae *and three dicot species. It was found that compositional patterns in the dicot species resembled those of cold-blooded vertebrates, while the grasses resembled warm-blooded vertebrates. Bimodality of GC_3 _distribution in grasses, and specifically in rice, was reported by Carels and Bernardi [3], Wang and Hickey [18] and Salinas *et al*. [12]. These authors explained the differences in codon usage among some rice genes by a rapid evolutionary increase in GC content. They gave two possible explanations for the observed bi-modality: (1) positive Darwinian selection, acting at the level of translational efficiency; and (2) neutral mutational bias.

Several characteristics related to high GC_3 _genes have been observed to date. Duret *et al*. [2] examined vertebrate sequences and described two properties of high-GC_3 _genes: the proteins are generally shorter, and introns are either absent or short in comparison to low-GC_3 _genes. Carels and Bernardi [3] compared genes in plants with generally high GC content to those with generally lower GC content. Although the differences were most prominent in *Gramineae*, they observed that other families of plants including dicots (e.g. *Brassicaceae *and *Fabaceae*) could be segregated by GC distribution. They also observed the tendency towards short or no introns in GC-rich genes and identified a correlation between GC content, intron size and location among homologs across species. Duret *et al*. [17] reported a small correlation between GC_3 _and the general GC richness of the surrounding >10 kb of genomic sequence. The relationship between gene length and GC_3 _for many organisms has been analyzed in a number of publications during the last decade [18-21]. Gene lengths in *C. elegans, D. melanogaster, A. thaliana *[19] and *O. sativa *[18] are negatively associated with GC_3_. Shorter genes in bacteria tend to have more variable expression levels, and selective pressure on codon usage is also higher in shorter genes [22]. It was recently demostrated that corn genes with high GC_3 _tend to be mono-exonic [23]. It has been reported that shorter and intron-poor genes have either stronger [24-26] or more variable [27,28] expression levels because introns can delay regulatory responses and are selected against in genes whose transcripts require rapid adjustment for survival of environmental challenges [28]. Ren *et al*. [25] showed opposite trends in plant and animal genomes; highly expressed genes tend to be longer in plants and shorter in animals. A recent paper by Jeffares *et al*. [28] proposed a reconciliation of these observations: both plants and animals show consistent inverse relationships between intron density (defined as intron number/unspliced transcript length) and rapid regulation (measured as the fastest rate of change of gene expression intensity in a time course experiment).

An influence of translation on codon bias has been proposed on the basis of increased hydrogen bonding and hence strength of G-C pairing in contrast to A-T pairing. This increased pairing may improve transcript stability at the mRNA level or improve the speed or fidelity of translation, thereby improving protein production, as has been shown in a number of species including bacteria and some eukaryotes [29]. This is supported by the analysis of Campbell and Gowri [1], who studied codon usage in plants and found two groups of genes that had preferences for GC-ending codons in monocots but not dicots. Additionally, Jabbari *et al*. [30] found a correlation between high-GC genes and amino acid hydropathy. However, Wang and Hickey [18] used concordance analysis of synonymous and non-synonymous differences to show that the primary effect is not at the codon or protein level.

Several groups [3,14,18,22,31] have suggested that the effect of high or low GC_3 _may be at the level of transcription. The generally shorter introns and coding sequences of high-GC_3 _genes led Carels and Bernardi [3] to suggest that selective pressure has driven housekeeping and non-regulated genes to higher GC contents while the longer AT-rich genes have been maintained to provide more opportunity for regulation and alternative splicing. Clay *et al*. [14] looked at CpG islands upstream of GC-rich and GC-poor transcripts and found little correlation. Nevertheless, the observation of higher GC within the introns of GC_3 _transcripts as well as the 5' region, and the weak correlation between general genomic GC content and GC_3 _level, suggests that the transcriptional machinery may be involved.

Conflicting ideas about codon usage bias and expression levels have been published. Wang and Hickey [18] reported that codon bias is not correlated with gene expression. Using *S. cerevisiae *expression and sequence data, Dekker [32] showed that on average, GC-rich genes are significantly more transcriptionally active than AT-rich genes. A recent paper by Roymondal *et al*. [22] presented an expression measure of a gene, devised to predict the level of gene expression from relative codon bias. They suggested that since the bias is caused by the presence of optimal codons that are recognized by the most abundant tRNA species, the high-GC_3 _peak appears as a manifestation of natural selection acting in grasses and warm-blooded vertebrates. This process shapes the codon usage patterns for selected genes to gain optimal expression levels in response to changing environments. Roymondal *et al*. [22] mentioned that within any genome, codon bias tends to be much stronger in highly expressed genes.

Attempts have been made to discover an association between functional classes of genes and GC_3_. Carels and Bernardi [3] characterized the high GC-containing transcripts as housekeeping and photosynthetic. D'Onofrio *et al*. [33] found GC_3 _to be higher in genes involved with cellular metabolism and lower in those involved with information storage processing. These observations are consistent with previous studies of general GC contents of genes in arabidopsis [34].

The existence of a codon usage gradient along the coding regions was previously discussed by Hooper *et al*. [21], who outlined the possible advantages of a positive GC_3 _gradient. Based on an analysis of *E. coli *genes, the authors suggested that G_3_-containing codons may be translated more quickly and with lower error rate than other codons, thus avoiding congestion at the ribosomes because of a gradual increase of speed of translation along the gene. Wong *et al*. [35] discovered that in the plant kingdom, *O. sativa *genes are richer in GC at the 5' end than at the 3' end. This gradient and imbalance in nucleotide strand composition extends beyond the coding region; transcription start sites are characterized by a pronounced peak in CG-skew [36,37], and mRNAs tend to be purine-rich (A for low GC organisms and G for high GC organisms) [38,39]. Avoidance of unnecessary 'kissing interactions' between and within mRNAs was mentioned by Lao *et al*. [40] as a possible explanation for purine loading. Species adapted to hotter environments have stronger selection pressure towards purine loading since nucleic acids are "stickier" at high temperatures [40]. This effect is the most pronounced at the wobble position of codons.

Aside from transcriptional and translational influences, it is possible that the driver for differences in GC_3 _operates at a recombinational level. Gene duplication in the *Poaceae *has been mentioned as one possible explanation of GC_3 _bimodality [41]. The authors suggested that duplicated genes in *O. sativa *can be partitioned into 10 blocks by chromosomal location; these blocks have significantly different synonymous substitution rates (Ks). Wang *et al*. [41] found that Ks was negatively correlated with the GC content at the third position of codons (correlation coefficient -0.455) and that the bimodal distribution of Ks was split into two unimodal distributions corresponding to high- and low-GC_3 _genes. Related to this idea are advances in understanding of the accelerated evolutionary rates of some genes. Holmquist [42] described a model in which hybridization of similar genes during recombination resulted in a bias toward higher GC content in the recombined areas. Birdsell [43] demonstrated that recombination significantly increases GC_3 _in a selectively neutral manner; the GC-biased mismatch repair system evolved in various organisms as a response to AT mutational bias. Birdsell [43] suggested that unimodal low-GC_3 _species may have prevailing AT mutational bias, random fixation of the most common types, or mutation or absence of GC-biased gene conversion [44]. The authors hypothesized that recombination is more likely to occur within conserved and regulatory regions of the genome; therefore, introns, intergenic regions and pseudogenes tend to have lower GC contents than ORFs. Galtier *et al*. [45] noticed that GC-biased gene conversion, frequently accompanied by an increase in GC_3_, influenced the evolutionary trajectory of human proteins by promoting the fixation of deleterious AT→GC mutations. These observations raise the possibility that the high-GC_3 _class of genes might have appeared as a consequence of accelerated evolution.

With the increasing amount of genomic and transcript information available within the public databases as well as the improved understanding of gene conversion and gene regulatory mechanisms, we returned to the puzzle of GC_3 _bimodality in grasses in an effort to understand the significance of this phenomenon. We concentrate our discussion around *Oryza sativa *as it is one of the best-studied grass species at the genomic level.

## Results

### Gene classes in several organisms are readily identified by GC_3 _plots

We revisited the extent of variation of GC_3 _found in various species. In Figure 1 we have plotted the distributions of GC_3 _for 12 plant and animal species. Distributions of GC_3 _in *H. sapiens, O. sativa, C. reinhardtii*, and *Z. mays *are clearly bi-modal, *A. cepa, A. thaliana, G. max, S. cerevisiae *and *C. elegans *are unimodal, and *B*. *napus*, *D. rerio *and *M. musculus *have intermediate distributions. Uni- and bi-modality of GC_3 _distributions in various organisms have been reported previously [3,18,46] and our results are consistent with the earlier observations on the species tested.

### Isochores may not explain the presence of GC_3_-rich genes in grasses

Previous reports on GC_3_-rich genes have suggested that these are present in GC-rich regions of the genome, aka isochores [10,12,47]. The authors suggested that GC_3 _bimodality in grasses came about because these genes are located in regions of their respective genomes that differ in G+C content. Two decades of full genome sequencing and annotation of numerous plant genomes make it worthwhile to revisit the issue of codon usage in plants and plant isochore organization. In order to answer the question of isochores in grasses, we analyzed the GC contents of coding and non-coding sequences in *O. sativa*. Overall, the correlation of GC_3 _values between adjacent genes is 0.05, indicating that there is no significant clustering of these genes. We separated all mRNA-validated rice genes into two groups on the basis of GC_3 _content: the "low" group, where GC_3_<0.8, contains 11,608 genes; and the "high" group, where GC_3 _≥ 0.8, contains 4,889 genes. The choice of cut-off point between the two groups was based on the position of the lowest GC_3 _value between the two peaks. (This approach is different from the one outlined in [3] and [47], where the two classes were distinguished by overall GC content. In those two studies, the average GC_3 _contents were 0.89 in the high group and 0.69 in the low group). We analyzed the spatial distribution of genes with high GC_3 _values. Of the 4,889 genes in the high group, 3,661 are evenly distributed across the genome; 485 genes (out of for the remaining 1,228) occur in 36 clumps of 10 or more genes (Additional file 1: Supplementary Figure SF1 and Additional file 2: Supplementary Table ST1). Five of these clumps are likely to result from relatively recent gene duplication, since they consist of genes with identical PFAM annotations. From the analysis of seven animal species, Elhaik *et al*. [16] inferred that GC_3 _can only explain a very small proportion of the variation in GC content of long genomic sequences flanking the genes, and correlations between GC_3 _and GC in the flanking region decayed rapidly with distance from the gene. Accordingly, we examined 1,000 nucleotides upstream of the 16,497 rice genes and also found no significant correlation in GC content between the open reading frames and flanking regions. The GC contents of the high and low groups gave nearly identical unimodal bell-shaped frequency distributions centered at GC = 0.4. These results suggest an absence of isochore organization in the rice genome and indicate that the high-GC_3 _genes are not closely associated with GC-rich regions in rice.

### GC_3 _correlates with variability of gene expression

**Figure 2:**
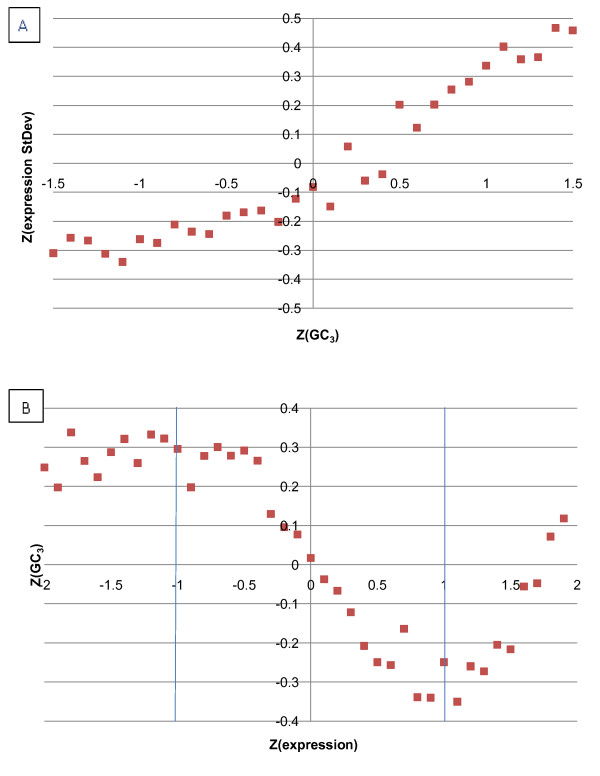
**Variability of gene expression in rice as a function of GC_3 _content in the CDS**. A: Rice expression variability as a function of GC_3 _frequency. X-axis: Standardized GC_3_, Y-axis: Standardized Expression Variability. B: GC_3 _content in rice genes as a function of gene expression. X-axis: Standardized Gene Expression, Y-axis: Standardized GC_3_. Number of genes per each data point for both plots is ~200, resulting in a standard error less than 0.05 for each data point in the graphs.

Previous reports have concluded that high-GC_3 _genes are associated with highly expressed transcripts [34]. We revisited this observation in rice by examining the expression levels of *O. sativa *genes. In order to dissect the pattern, we computed GC_3 _and the standard deviations of expression levels for 15,625 *O. sativa *genes across 106 series of gene expression measurements (see **Methods**). The standard deviations of gene expression and GC_3 _values were converted to standardized z-scores and plotted (Figure 2A). There is a strong positive correlation between the two measures: if we group genes by GC_3 _and compute the average z-score of standard deviation of expression for each group, the relationship can be interpolated using the linear regression equation y = 0.228x + 0.0294, R^2 ^= 0.7437. This shows that genes with higher values of GC_3 _have more variable expression profiles than genes with low GC_3 _values. We also plotted GC_3 _as a function of gene expression (Figure 2B). The relationship between average gene expression and GC_3 _is not as straightforward as between GC_3 _and variability of expression. It appears that for the majority (10,514) of genes with expression z-scores between -1 and 1, average expression level and GC_3 _are negatively correlated; for a subset of strongly expressed genes (2,224 genes with average standardized expression > 1), the relationship is positive; genes that are weakly expressed (2,887 genes with z-scores < -1) tend to have high GC_3 _and show no significant correlation between GC_3 _and expression. This observation may explain why conflicting results have been reported: weak, positive or negative correlations between codon usage and expression level [18,48,49]. The observed saddle-like pattern (Figure 2B) and the linear relationship between standard deviation of gene expression and GC_3 _(Figure 2A) may indicate the presence of distinct functional classes of genes in which the two quantities are differently related to each other. The discovery of relationships between expression levels and GC_3 _motivated us to analyze the promoters of well-annotated *O. sativa *genes.

### GC_3 _correlates with the presence of an upstream TATA box

**Figure 3:**
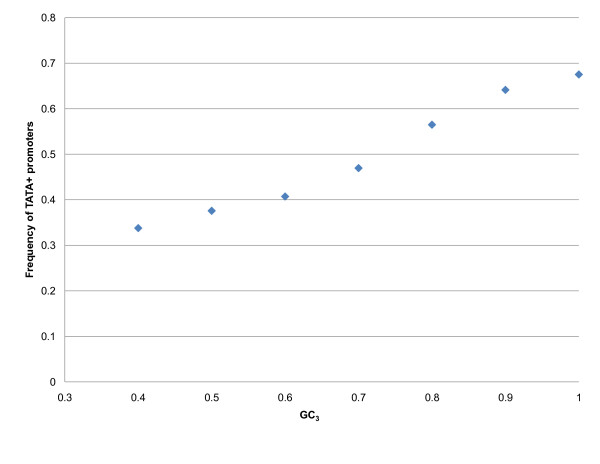
**Frequency of TATA+ promoters in rice as a function of GC_3 _content in the CDS**. GC_3 _is positively correlated with the presence of upstream TATA boxes. Each data point on this plot contains information from at least 700 genes.

For decades, it was believed that genes whose promoters contain TATA boxes (TATA+) are more highly expressed than those that do not (TATA-) [50]. Yang *et al*. [51] demonstrated that TATA- genes are frequently involved in "houskeeping" activities in the cell while TATA+ genes are often highly regulated by biotic or stress stimuli. In 2008, Moshonov *et al*. [52] reported that TATA+ genes are generally shorter than TATA- genes, and gene expression is influenced by a combination of core promoter type, presence of introns and gene size. It was recently shown by Troukhan *et al*. [53] that TATA+ promoters belong to genes with higher standard deviations of microarray intensity than TATA- promoters. Additionally, a saddle-like pattern similar to that in Figure 2B was observed when the frequency of TATA-boxes was plotted as a function of expression level. We have previously demonstrated [53] that different GO categories have different frequencies of TATA+ genes. For example, almost 60% of oxidative stress-related genes have TATA boxes, in sharp contrast to 20% of protein folding-related genes. Since expression variability and gene class appear to be correlated with codon usage, we decided to "complete the triangle" and calculate the frequency of TATA boxes in relation to the GC_3 _levels of genes. Approximately 30% of rice promoters in Osiris database contain a canonical TATA-box in the 50 nucleotides upstream of the transcription start site. Only 18% of rice genes with GC_3 _< 0.45 are equipped with TATA+ promoters as compared to 52% of those with GC_3 _> 0.95 (Figure 3, Additional File 1: Supplementary Figure SF2). We hypothesize that the presence of TATA in the promoter establishes a conformational framework for transcription factors that facilitates reliable and timely transcription initiation. Therefore, stress-response genes tend to have a higher frequency of TATA-boxes. An additional GC pair makes translation more efficient and better coordinated with transcription [21]. A stress-related protein has to be produced quickly and reliably at the onset of a stress condition. Therefore, insertion of a TATA box in the promoter sequence and elevated GC_3 _frequency ensure rapid production without altering the amino acid sequence.

### Number of expressed paralogs and orthologs is negatively associated with GC_3_

**Figure 4:**
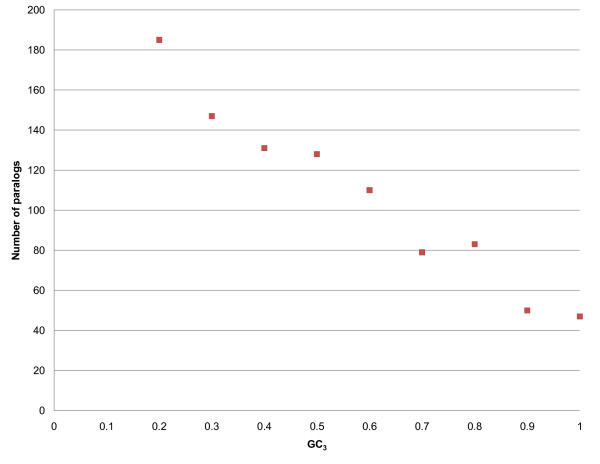
**Number of expressed paralogs and GC_3_**. Number of expressed paralogs in the rice genome is negatively associated with GC_3_.

Using the same logic, it is easy to explain why the number of gene paralogs is negatively correlated with GC_3 _(Figure 4). If a genome has multiple copies of a gene (ideally equipped with the same promoter machinery), any one of these copies may be transcribed and translated to obtain the required protein. On the other hand, unique copies of genes have to be optimized transcriptionally and translationally to ensure protein production. These ideas were first formulated almost a decade ago, when Hooper *et al*. [21] suggested that there is positive selection on codons that are translated more efficiently, either more quickly or more accurately. Similarly, considering the specificity of a gene in an organism, highly species-specific genes (those lacking orthologs in other species) tend to possess high GC_3_: 44% of *O. sativa*-specific genes have GC_3 _≥ 0.8 as compared to 29% for all genes. This is consistent with the assumption that equilibrium is reached at the high AT end of the spectrum, and unless there is evolutionary pressure to maintain high GC content, a gene will become AT-rich. The requirement for protein sequence conservation explains why the effect is most pronounced at the third position of a codon. We have to point out that our earlier observation [23], that horizontal gene transfer may explain GC_3 _richness in a certain fraction of grass genes, is not applicable to all genes in the high-GC_3 _class. We found that among the expressed genes (defined by presence of mRNA) in rice, 46% of those in the high-GC_3 _class have orthologs in *A. thaliana*, as compared to 60% of the low-GC_3 _genes. However, if we consider all rice genes regardless of expression level (41,129 excluding transposons), we find that 65% of the high-GC_3 _class and 57% of the low-GC_3 _class have orthologs in *A. thaliana*. This means that GC_3 _content affects the expression pattern. Many high-GC_3 _genes with homologs in arabidopsis are not expressed in rice, probably because they have been silenced by methylation and will be activated only under extremely rare stress conditions.

### GC_3 _is negatively correlated with gene length and intron density

Genes in the middle of the GC_3 _spectrum (0.4<GC_3_<0.7) have a negative correlation with ORF length (Pearson's correlation coefficient = -0.3), whereas for genes in the high GC_3 _class and for those with GC_3_<0.4, it is approximately 0. As was previously observed [28], variability of gene expression is negatively correlated with intron density. We computed Pearson's correlation coefficient between GC_3 _and intron density for *O. sativa *and *S. bicolor*: for both grass species it is approximately -0.3 (Additional File 1: Supplementary Figure SF3). Genes with high GC_3 _tend to be mono-exonic [23]. This is consistent with our observation of a positive relationship between gene expression variability and GC_3_. On the basis of this evidence, we suggest that rapidly evolving genes are shorter, have more variable expression and are GC_3_-rich. More "evolutionarily stable" genes tend to accumulate introns and increase the ORF length.

### Gradient of codon usage along the gene

**Figure 5:**
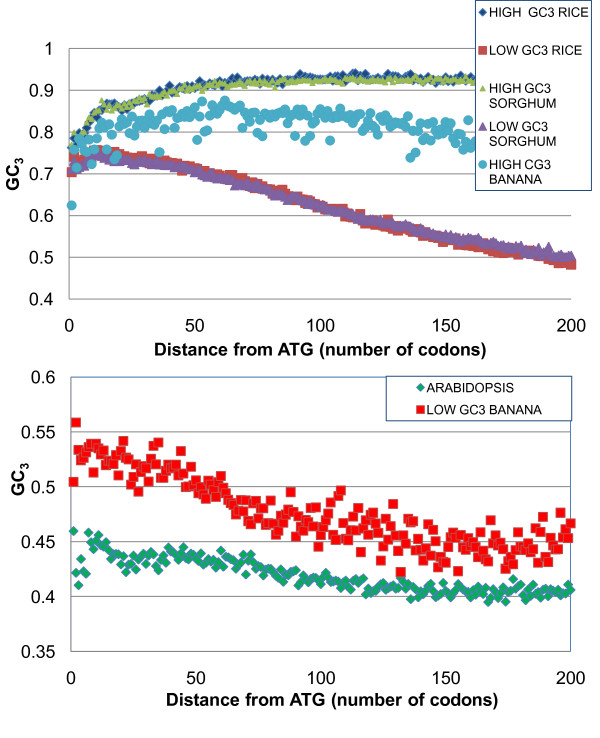
**GC_3 _gradient from 5' to 3' ends of coding regions**. At the 5' end of the open reading frame, high GC_3 _genes of rice, sorghum, and banana have a slight positive gradient, whereas low GC_3 _genes in arabidopsis, rice, sorghum, and banana become more AT_3_-rich towards the 3' end.

**Figure 6:**
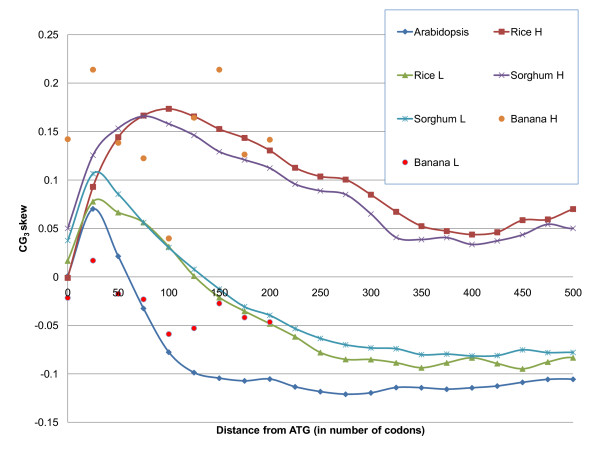
**CG_3 _skew in plant coding regions**.  stratified by GC_3 _classes (High (H): GC_3 _≥ 0.8 and Low (L): GC_3_<0.8) for coding regions of arabidopsis, rice, sorghum, and banana. Genes are aligned by ATG, and frequencies of nucleotides are computed in a sliding window of 50 nucleotides.

Analysis of coding sequences in several organisms has indicated a gradient in codon usage from the 5' to 3' ends of genes [4,21,35]. Based on analysis of *E. coli *genes, Hooper *et al*. [21] suggested that G_3_-containing codons may be translated with lower error rate and more quickly than other codons, thus avoiding congestion of ribosomes owing to a gradual increase of speed of translation along the gene. According to Wong *et al*. [35], *O. sativa *genes are richer in GC at the 5' end than at the 3' end. *A. thaliana *shows no such trend in GC usage. Lescot *et al*. [4] reported that there are two distinct classes of genes in the *Musa acuminata *(banana) genome: those with arabidopsis-like and those with rice-like gradients. *M. acuminata *is a monocot plant that belongs to the same order of commelinids as *Poaceae*. Analysis of GC_3 _distribution in the members of the *Zingiberales *order, banana, ginger and turmeric, indicates the possibility of bimodality (see [4] and Figure 5 in this work). Unfortunately, the number of currently sequenced CDSs for the *Zingiberales *order is too small for conclusive statistical analysis. For *O. sativa *genes from the high and low GC_3 _classes, we computed the  and found that genes in the high-GC_3 _class have a slight preference for C_3 _(therefore, more G_3 _in mRNA) and the low-GC_3 _class have a slight preference for G_3_. The overall correlation coefficient between GC_3 _skew and GC_3 _content is approximately 0.45. We plotted GC_3 _skew as a function of the number of codons from ATG (Figure 6). Genes from the high-GC_3 _peak in rice, sorghum and, probably, banana have a preference for C_3 _over G_3_. This preference initially increases from 5' to 3' and then peaks and levels off at *CG*_3 _skew ≈ 0.05. Genes in the low-GC_3 _class have a similar tendency in the first 50 or so codons but then show a strong preference for G_3 _over C_3_, CG_3 _skew ≈ -0.1. Since the low-GC_3 _class is approximately twice as abundant, when genes from the high- and low-GC_3 _classes are considered together, the prevailing tendency is for GC to decrease toward the 3' end, as observed by Lescot *et al*. [4]. If the translation efficiency explanation for genes [21] carries over to eukaryotes, genes with positive GC_3 _skew have more G_3 _in mRNA and therefore more optimal codons. Following this explanation, we hypothesize that genes in the high-GC_3 _class must be more important for the well-being of an organism than genes from the low-GC_3 _class.

Codon usage and gene classes

The first two nucleotides in a codon are more reflective of gene function than the third one. Using coding sequences of *O. sativa*, we computed average GC_3 _and GC_12 _for GO and FPAM annotations. The coefficient of variation for GC_12 _is approximately three times smaller than the coefficient of variation for GC_3_. However, the third position in the codon also affects gene function. Liu *et al*. [54] demonstrated that synonymous codon usage and gene function are strongly correlated in *O. sativa*; they found that genes involved in metabolic processes have a preference for C or G in the third position of a codon. Different PFAM families show affinity for high- or low-GC_3 _classes. For example, *O. sativa *genes annotated as "expressed proteins" are more prevalent in the low class (22% vs. 33%) and alpha-expansins are more prevalent in the high group (relative abundance is 46). Details are given in the Supplementary data (Additional File 2: Supplementary Table ST2). It appears that GC_3 _increase tends to co-evolve in some PFAM families of grass genes across multiple organisms. The distribution of GC_3 _in histone, ribosomal and chrolophyll a-b binding protein coding genes are very similar for rice and corn. In both organisms, 80% of chrolophyll a-b binding proteins have GC_3_>0.85, ribosomal proteins are approximately normally distributed around GC_3 _= 0.65, and 60% of all histones have GC_3_>0.75. Another way to look at the relationship between gene category and GC_3 _is by considering GO annotation (see Additional File 2: Supplementary Tables ST3-ST7). *D. rerio, M. musculus, H. sapiens, C. reinhardtii, O. sativa *and *Z. mays *have higher GC_3 _values than *A. thaliana *and we were curious to see if GC_3 _is consistent between these organisms and GO categories. The high-GC_3 _species also have consistently higher GC_3 _values for genes from the following GO classes: electron transport or energy pathways, response to abiotic or biotic stimuli, response to stress, transcription and signal transduction. Therefore, we conclude that certain classes of genes are characterized by high GC_3 _values across kingdoms.

### GC_3 _in CDS and GC genomic context are not correlated

**Figure 7:**
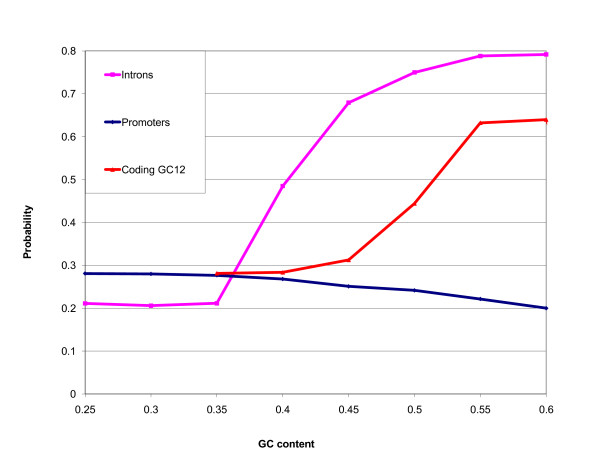
**Probability of being in the high GC_3 _peak as a function of GC content of introns, coding GC_12 _and promoters (*O. sativa*)**. GC content of introns seems to have the most striking effect on GC_3 _content in coding regions.

Using the genome of *O. sativa*, we computed the probability of a rice gene belonging to the high-GC_3 _peak on the basis of the GC content of its promoter, coding GC_12 _and introns (Figure 7). The MSU collection of rice upstream sequences contained 66,710 1-kb long sequences, of which we retained 16,497 corresponding to our curated set of ORFs. MSU's collection of introns contained 252,431 sequences. We eliminated introns containing blast hits to *O. sativa *ESTs. The resulting set contained 12,571 loci with introns. The dividing line between high and low GC_3 _was set at 0.8. We observed that of these three gene parts, the GC content of introns seems to have the most striking effect on GC_3 _content in coding regions. The reduced influence of GC_12 _can be explained by constraints imposed by protein sequence conservation. There is also no dependency between GC_3 _and the GC content of 1000 nucleotides taken from the 3' flanking region (it has a negligible correlation coefficient of -0.02; data not shown). Introns are generally AT-rich, with only a small fraction having high GC values. To test the statistical significance of this effect, we used our curated set of 12,571 genes that have introns and performed a chi-squared test. The resulting p-value was 8.8 × 10^-12^, so the effect is highly significant. Similar results were obtained for another grass, *S. bicolor *(data not shown). The sharp increase in probability of being in the high-GC_3 _class for genes with GC-rich introns suggests that the appearance of high-GC_3 _genes in grasses is unlikely to be linked to a translational mechanism.

### High-GC_3 _genes have more targets for methylation

**Figure 8:**
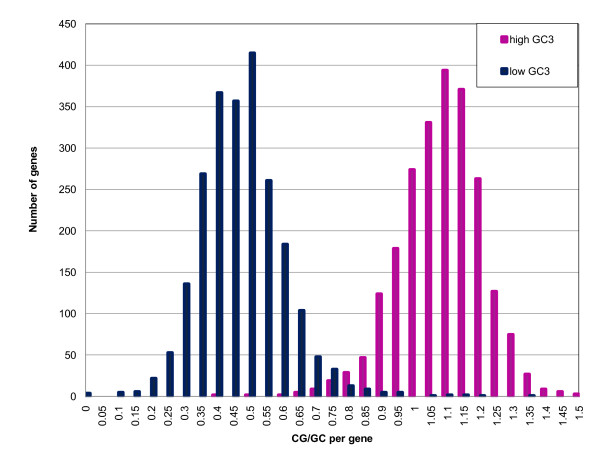
**Distribution of ratio of di-nucleotide frequencies CG to GC in *O. sativa *CDSs**. High-GC_3 _genes provide more targets for methylation than low-GC_3 _genes.

**Figure 9:**
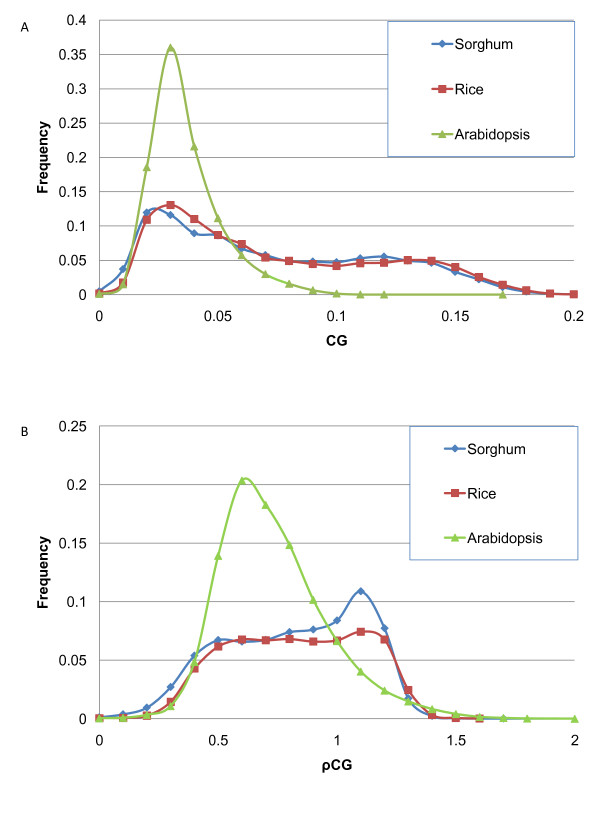
**Distribution of frequency (A) and relative abundance (B) of CG for *A. thaliana, O. sativa *and *S. bicolor***.

Kalisz and Purugganan [55] proposed that GC content may affect gene transcription. They observed that naturally-occurring variations of gene methylation (termed epialleles) can influence the level of gene expression and produce novel phenotypes. The authors found that methylated epialleles in plants are associated with organ specificity and stress response. Salinas *et al*. [12] reported that GC-rich genes provide more targets for methylation. The estimates were made using 5mC quantification by HPLC and methylation over available di- and tri-nucleotide targets of methyltransferases. A recent paper by Stayssman *et al*. [56] points out that housekeeping genes are not methylated and are therefore constantly available for transcription, whereas tissue-specific genes are methylated and generally inactivated. Stayssman et al. [56] observed a relatively large number of CpG islands that seem to be fully methylated in most cell types but unmethylated in a single tissue. These regions are initially unmethylated, and then they undergo *de novo *methylation in all somatic cells during development. Methylation may be involved in repression of the genes in these islands. These observations are consistent with our findings that genes in the high-GC_3 _peak tend to have a higher frequency of methylatable CG dinucleotides (discussed below, Figure 8) and are more differentially expressed under various stresses, among tissue types and among developmental stages. In order to illustrate this fact better, we selected 2,300 genes from the high-GC_3 _peak in rice and compared them to 2,300 genes with the lowest GC_3 _contents. For these genes, we computed CG and GC frequencies in CDSs (relative frequencies CG/GC are shown in Figure 8 and raw frequencies are in Additional File 1: Supplementary Figure SF4). Apparently, a high-GC_3 _peak is characterized by an increased preference for CG over GC and a low-GC_3 _class favors GC over CG (the peak is centered at ~0.5). We suggest that a regulatory mechanism acts differentially on high- and low-GC_3 _genes. One may wonder whether these pronounced differences are purely statistical rather than biological, as dinucleotide frequencies depend on overall nucleotide composition. Therefore, we examined the differences using relative abundance values (defined in **Methods**) that account for background nucleotide distribution. Additional analysis was needed to establish the extent of this effect. We computed di- and tri-nucleotide frequencies and relative abundance values (Figure 9) for coding regions of *A. thaliana, S. bicolor *and *O. sativa*. Frequencies and relative abundance values of CG in rice and sorghum have bimodal distributions, while tri-nucleotide frequencies and relative abundance values are unimodal. Although the frequencies of trinucleotides of the type CWG, where W stands for A or T, differ among the organisms studied, the distribution of relative abundance of CWG is nearly identical for *S. bicolor, O. sativa *and *A. thaliana*.

**Figure 10:**
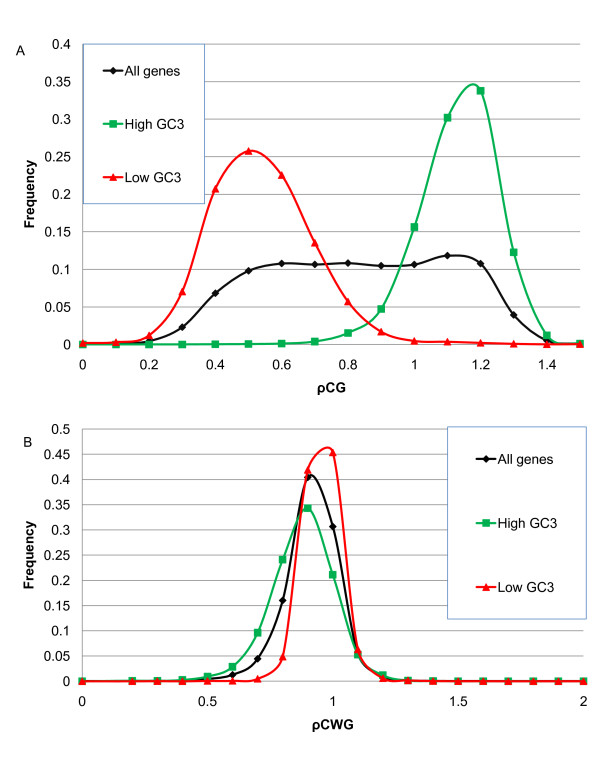
**Distribution of relative abundance of CG (A) and CWG (B) at all positions for all genes and in the high and low GC_3 _classes of *O. sativa***.

**Figure 11:**
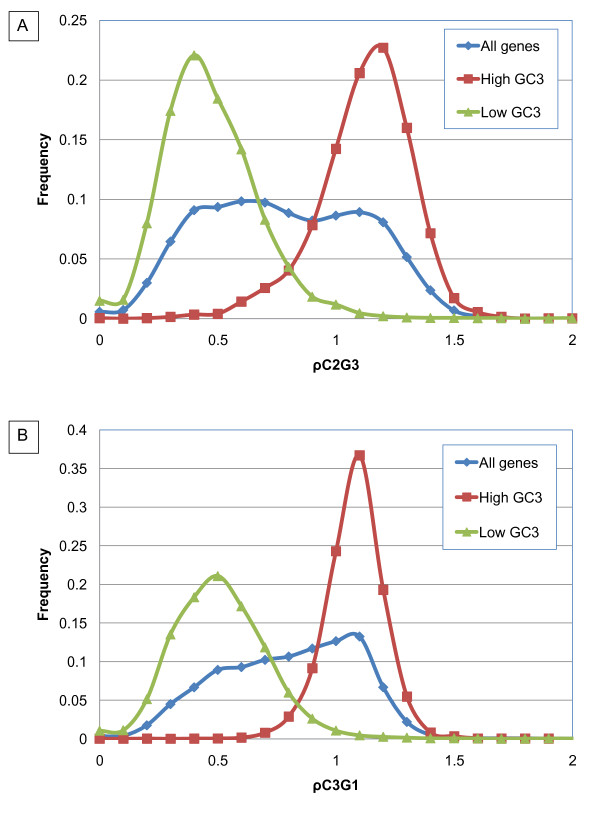
**Distribution of relative abundance ρC_2 _G_3 _(A) and ρC_3 _G_1_(B) at the wobble position for *O. sativa***.

The bimodality of genome signature distribution indicates the presence of hidden covariates. Tran *et al*. [57] reported that cytosine DNA methylation in plants is found predominantly in transposable elements and repetitive DNA, where methylcytosines are typically found in CG and CWG. Methylation of CG and CWG sites is maintained by different mechanisms: CG sites are maintained by a plant homolog of mammalian *Dnmt1 *acting on hemi-methylated DNA after replication [57] whereas methylation of CNG sites (N is any nucleotide) is maintained by other mechanisms. Methylation of Cs that are followed by Gs is a regulatory strategy employed by some eukaryotes [58]. Figure 10 shows that the high-GC_3 _class has a significantly greater proportion of the dinucleotide CG than the low-GC_3 _class and that there is no significant difference in the relative abundance of CWG between the two classes of genes. Since there is evolutionary pressure to keep the first and second nucleotides in the codon intact, we analyzed position-specific genomic signatures ρC_3_G_1 _and ρC_2_G_3 _(Figure 11) and position-specific frequencies of nucleotides in the third position, conditional on having cytosine in the second position. For the high-GC_3 _class of genes there is a slight preference for G in the third position: P(G_3_|C_2_)/P(C_3_|C_2_) = 1.12. For the low-GC_3 _genes, there is a more pronounced difference between conditional probabilities, and the CG combination was the least favored for rice: P(G_3_|C_2_)/P(C_3_|C_2_) = 0.6. Another possibility for obtaining CG dinucleotides without altering the amino acid sequence is to place C in the third position of a codon that is immediately followed by a codon starting with guanine. Genes from the low GC_3_-peak have cytosine in the third position of the previous codon less frequently than high GC_3 _genes. For the high-GC_3 _genes, there is a preference for C in the third position: P(C_3_|G_1_)/P(G_3_|G_1_) = 1.52. For the low GC_3 _genes, there is an opposite trend: P(C_3_|G_1_)/P(G_3_|G_1_) = 0.45. A similar pattern was found when we examined sequences of *S. bicolor*. For comparison, we computed position-specific enrichments for *A. thaliana*. Since GC_3 _has a unimodal distribution, we observe no significant differences between high-GC_3 _and low-GC_3 _genes in arabidopsis (see Additional File 1: Supplementary Figure SF5).

**Figure 12:**
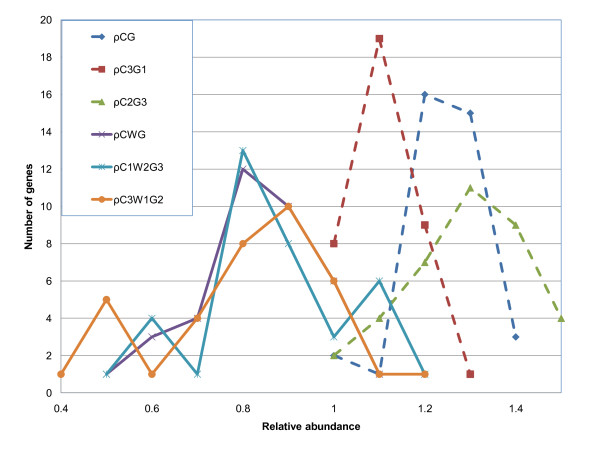
**Distribution of position-specific relative abundance for αβ-hydrolase, *O. sativa***.

We analyzed protein families containing genes of *O. sativa *that are either enriched in CG and depleted in CWG or enriched in CWG and depleted in CG. Figure 12 shows the distribution of position-specific relative abundance for αβ-hydrolase-3 proteins of *O. sativa*; αβ-hydrolase-3 represents a family of proteins that is enriched in CG and depleted in CWG. On the other side of the enrichment spectrum are genes containing HEAT repeats (Additional File 1: Supplementary Figure SF6); they are depleted in CG and the relative tri-nucleotide abundance is ~1. These proteins function as protein-protein interaction surfaces; many HEAT repeat-containing proteins are involved in intracellular transport processes. Protein kinases (Additional file 1, Supplementary Figure SF7) participate in many processes such as cell division, proliferation, apoptosis and differentiation. This diversity may explain the wide bimodal distribution of dinucleotide abundance values for protein kinases. In analyzing the relative abundances of the dinucleotide CG in *O. sativa *and *S. bicolor *we noticed that different Gene Ontology categories and Protein Families have preferences for certain nucleotide compositions, and these preferences are consistent between the two organisms. Conservation is higher for certain gene categories than for others: for example, genes that belong to the "transcription regulation activity" GO function category have a correlation coefficient of 0.91 between rice and sorghum, and 0.46 between rice and arabidopsis. Genes that have "motor activity" function have a correlation coefficient of 0.36 between rice and sorghum, and 0.13 between rice and arabidopsis. These observations support the earlier suggestion of Pradhan *et al*. [59] that the action and control of CG and CWG methyltransferases might be different and that CG and CWG methylation may serve different biological functions.

### High-GC_3 _genes and GC-biased gene conversion

Many previous studies have demonstrated a significant association between GC_3 _and recombination rate across different plant and animal species [41,42,44,45,60-62]. The conclusion is that high GC_3 _content in an organism indicates a recombining genome. Similarly, the presence of two distinct GC classes of genes may suggest the existence of recombining and non-recombining regions within that genome. To support this hypothesis, we computed the mutation rates of rice genes (see **Methods**). For our curated dataset of 16 K rice genes, we found a positive correlation between the density of SNPs per 1000 nucleotides and GC_3 _(R^2 ^= 0.71, SNP = 1.114+0.583GC_3_). Association with overall GC content is much weaker, R^2 ^= 0.32. Therefore, we conclude that high-GC_3 _genes accumulate more mutations and are located in the highly recombining regions of the rice genome.

### Importance of GC_3_

Analysis of gene-specific codon usage bias shows that GC_3 _is the major characteristic of codon utilization in *Poaceae*. In order to demonstrate this, we used Principal Component Analysis (PCA) to find a basis for the space of codon vectors. Approximately 50% of the variance in codon usage is explained by the first principal component; this component has an almost perfect negative correlation (-0.98) with GC_3_. The remaining components contribute at most 4% each to the variance; the second principal component is weakly correlated to GC_3 _skew.

## Discussion

Deviations from unimodal bell-shaped distributions of GC_3 _appear in many species, but grasses have very pronounced bimodal distributions (Figure 1, Additional File 1: Supplementary SF8 and SF9). Bimodality in warm-blooded vertebrates can be explained by the presence of isochores. Although there are many similarities between genes in high-GC human isochores and high-GC_3 _genes in grasses, the isochore hypothesis does not fully explain the existence of high-GC_3 _genes in grasses: first, there is no correlation between ORFs and the flanking regions; second, most species with isochores do not have a high-GC_3 _peak. Possible causes of bimodality may be elucidated by comparing genes in the high- and low-GC_3 _classes. These classes differ in nucleotide composition and composition gradients along coding regions. High-GC_3 _class genes have a significantly higher frequency of CG dinucleotides (potential targets for methylation); therefore, there is an additional regulatory mechanism for high-GC_3 _genes. Springer *et al*. [63] reported that out of eight classes of methyl-CpG-binding domain proteins present in dicots, only six exist in monocots, suggesting a difference between dicots and monocots in silencing of methylated genes.

Two competing processes may affect the frequency of methylation targets: the GC-based mismatch repair mechanism and AT-biased mutational pressure. In recombining organisms (e.g., grasses and warm-blooded vertebrates), the GC content of coding and regulatory regions is enhanced because of the action of the GC-based mismatch repair mechanism; this effect is especially pronounced for GC_3 _[43]. Recombination has been shown to be a driving force for the increase in GC_3 _in many organisms [64]. Repair (recombination) happens all over the genome with a certain precision, leading to an increase in GC. If repair did not occur in defence-related genes, the organism may fail to survive or to reproduce. If repair did not happen in less important genes (and, consequently, their GC content remained the same), it may not be detrimental to the organism. AT-biased mutational pressure, resulting from cytosine deamination [65] or oxidative damage to C and G bases [66], counteracts the influence of recombination; and in most asexually-reproducing species and self-pollinating plants, AT bias is the winning process. Our analysis from aligning *indica *and *japonica*, as well as earlier publications [43], indicate that genomic regions under higher selective pressure are more frequently recombining and therefore increase their GC_3 _content. This mechanism may explain the pronounced differences in GC_3 _between *A. thaliana *and its closest relatives. Comparison of the nucleotide compositions of coding regions in *A. thaliana, R. sativus, B. rapa, and B. napus *reveals that the GC_3 _values of *R. sativus, B. rapa*, and *B. napus *genes are on average 0.05 higher than those of the corresponding *A. thaliana *orthologs [67]. An important difference between *A. thaliana *and *Brassica *and *Raphanus *is that the latter two genera are self-incompatible, whereas *A. thaliana *is self-pollinating. Self-pollination in arabidopsis keeps its recombination rates low and thus reduces the GC_3 _content of its genes. Self-pollination is also reported in some grasses such as wheat, barley and oats. Analysis of recombination in wheat [68] showed that the genome contains areas of high and low recombination. Grasses have an efficient reproductive mechanism and high genetic variability that enables them to adapt to different climates and soil types [69,70]. We hypothesize that since self-pollination generally lowers recombination rates, evolutionary pressure will selectively maintain high recombination rates for some genes. Analysis of highly recombinogenic genomic regions of wheat, barley, maize and oat identified several genes of agronomic importance in these regions (including resistance genes against obligate biotrophs and genes encoding seed storage proteins) [69]. In addition to the methylation-driven growth of high-GC_3_, we hypothesize that developing GC_3 _richness in some genes may, if it is not balanced by AT-bias, work as a feed-forward mechanism. Once it appears in genes under selective pressure, it provides additional transcriptional advantage. GC pairs differ from AT pairs since guanine binds to cytosine with three hydrogen bonds, while adenine forms only two bonds with thymine. This additional hydrogen bond makes GC pairs more stable and GC-rich genes will have different biochemical properties from AT-rich genes. When an AT pair is replaced by a GC pair in the third position of a codon, the protein sequence remains unchanged but an additional hydrogen bond is introduced. This additional bond can make transcription more efficient and reliable, change the array of RNA binding proteins, or significantly alter the three-dimensional folding of the messenger RNA. In this case, those plant species that thrive and adapt successfully to harsh environments demonstrate a strong preference for GC_3 _in the third position of the codon.

High GC_3 _content provides more targets for methylation. The correlation between methylation and GC_3 _is supported by Stayssman *et al*. [56], who reported a positive correlation between methylation of internal unmethylated regions and expression of the host gene. In this paper we have demonstrated a positive correlation between GC_3 _and variability of gene expression; we also found that high-GC_3 _genes are more enriched in CG than the low-GC_3 _class. Therefore, GC_3 _classes provide more targets for *de novo *methylation, which can serve as an additional mechanism of transcriptional regulation and affect the variability of gene expression. Additional transcriptional regulation makes species more adaptable to external stresses.

Grasses have undergone several genome duplications. Genomic regions varied in their recombination rates and GC_3 _contents. Since high GC_3 _content in a gene provided an evolutionary advantage, this was frequently the sole copy retained in grasses. This may explain why genes in the high-GC_3 _class frequently lack paralogs. High-GC_3 _genes provide an evolutionary advantage owing to their optimized codon usage and to the existence of methylation targets allowing for an additional mechanism of transcriptional regulation. Therefore, the high-GC_3 _class of genes has been maintained in grasses for generations.

## Conclusions

In this paper we combine a variety of prior observations and insights on GC_3 _biology with new observations using larger genome data sets to establish a unifying framework of hypotheses to explain all the available data fully. This framework consists of evolutionary forces and sexual reproduction patterns to justify a wide variety of observed codon usage patterns in plants and animals. These evolutionary forces are realized through introducing new mutations during meiotic recombination and fixation with the help of DNA methylation and transcriptional mechanisms. The presence of GC_3_-rich genes is not likely to be a consequence of chromosomal isochores or horizontal gene transfer. Regardless of their initial origin, high-GC_3 _genes in recombining species possessed a self-maintaining mechanism that over time could only increase their drift towards even higher GC_3 _values. This uncompensated drift may explain the pronounced bimodality of some rapidly-evolving species. Competing forces acting in grasses make GC_3 _distribution distinctly bimodal; genes in the high-GC_3 _class are more transcriptionally regulated, provide more targets for methylation and accumulate more mutations than genes in the low-GC_3 _class.

## Methods

### Data sources

In our analysis, we concentrated on those plant species that benefit from complete sets of full-length cDNAs and sequenced (complete or nearly complete) genomic data. We used the following species: *O. sativa, S. bicolor, A. thaliana, C. reinhardtii, Z. mays, D. rerio, M. musculus and H. sapiens*. *O. sativa *genes and genomic sequences were downloaded from the Rice Genome Annotation project[71]; after exclusion of all transposon-like genes and genes without full-length cDNA support we obtained a final set of 16,497 genes. Rice promoter sequences were downloaded from the Osiris database [72]; positions of Transcription Start Sites were refined using the TSSer algorithm [53]. Rice microarray data were obtained from NCBI, Gene Expression Omnibus, platform GPL2025. We used two measures of expression: average intensity and standard deviation across 106 series of gene expression measurements. We used the recently published sequence and annotation data from the Joint Genome Institute for *C. reinhardtii *and *S. bicolor *(27,640), released 08/28/2008 [73] and 10/28/2008 [74] respectively. *A. thaliana *genes (27,741) were downloaded from The Arabidopsis Information Resource. Collections of *D. rerio, M. musculus and H. sapiens *sequences were taken from NCBI. *Z. mays *sequences were obtained from J. Craig Venter Institute. The remainder of the plant transcripts for the *Poaceae *family (aka grasses) were downloaded from TIGR Plant Transcript Assemblies[75]. We used the frequency of single nucleotide polymorphisms per 1-kb gene length, obtained from the Plant Genome Mapping Laboratory, University of Georgia [76], as a crude proxy for the local recombination rate in rice. Supplementary figures and tables are available at http://model.research.glam.ac.uk/projects/glacombio/GC3/.

### Calculation of z-scores

For each gene, GC_3 _values and the standard deviation of log-transformed gene expression values were computed across all experiments. Genome-wide distributions of both GC_3 _and gene expression are approximately normal. For each of these measures, the parameters μ (mean) and σ (standard deviation) of the corresponding normal distributions were determined. The standard deviations of gene expression and GC_3 _values were converted to z-scores, , and the standardized scores were plotted.

### Calculation of relative abundance

Relative abundance was calculated according to [77], in which it was observed that the profiles of relative dinucleotide abundance values (genome signatures) are equivalent to the "general design" of organisms, and closely-related species have similar genome signatures. The computational formulae for di- and tri-nucleotide relative abundance values are , where N stands for any nucleotide and W denotes A or T. As demonstrated by [78], the ratio of observed to expected CpG frequency underestimates the real CpG deficiency in GC-rich sequences: because the formula is non-linear, an identical fraction of mutated CpG in high- and low-GC classes of genes results in artificially higher values of ρ_*CG *_for the former than the latter. The authors suggested the use of a threshold of ρ_*CG *_as a function of G+C frequency to assess the presence of unmethylated sites, which can be calculated using the following formula: . In order to take the influence of this mathematical artifact into account in addition to the original relative abundance values, we also considered GC-corrected values defined as .

### Principal Component Analysis

Principal Component Analysis (PCA) involves a mathematical procedure that transforms a number of possibly correlated variables into a smaller number of uncorrelated variables called principal components. The data are represented in a new coordinate system such that the greatest variance of the data lies on the first principal component, the second greatest variance on the second coordinate, and so on [79]. Our approach was generally similar to that of Chen *et al*. [80]: for each gene *i *of *O. sativa *we calculated codon frequency *c*_*i*, *m*(*w*)_, where *m(w) *stands for *w*^*th *^codon for amino acid *m*, and applied PCA (using the *princomp *function in R).

## Authors' contributions

TT developed algorithms, conducted data analysis, interpreted the results and prepared the manuscript. NA generated hypotheses and was involved in data analysis and manuscript preparation. JB participated in the interpretation of data, discussions around the biological importance of observations and writing and editing of the manuscript. KAF was involved in all aspects of the analysis of the data and preparation of the manuscript. All authors read and approved the final manuscript.

## Authors' information

TT received her PhD in Applied Mathematics from the University of Southern California. Currently she is a Senior Lecturer in Statistics, University of Glamorgan, Wales. Prior to accepting this post she worked as a computational scientist for the biotechnology company Ceres, Loyola Marymount University and Georgia Institute of Technology. TT developed algorithms for analyzing gene expression analysis, discovering promoter motifs and genome annotation.

NA is a Senior Computational Scientist at Ceres. He received his PhD in Molecular Biology, VNII Genetika, Russia. He was a postdoctoral researcher in Kyoto University, Japan and later at NCI/NIH, and then a computational scientist at Amgen. He has done computational work on discovery of promoter motifs, protein structure, fold recognition and lead discovery.

JB earned his PhD in Molecular Biology and Genetics from the University of Pennsylvania and his BS in Computer Science from the University of Wisconsin. He has long-standing interest in genomics and has been leading bioinformatics and informatics efforts at the Human Genome Sequencing Center (Baylor College of Medicine), a biopharmaceutical company (UCB Pharma), and most recently at an agricultural biotechnology company (Ceres, Inc.)

KAF received his PhD in Genetics from Ohio State University. Upon graduation, he held positions in two companies, and later moved to the Dept of Plant Sciences at the University of Arizona. After accepting a position to start up a genomics company, Ceres, in 1997, KAF led the company's sequencing strategy, which resulted in the largest number of plant cDNAs that had ever been sequenced. Working with computational biologists at Ceres, including the three co-authors on this paper, he helped use these cDNAs to advance our understanding of plant transcriptomes. Currently KAF is the Director of the School of Plant Sciences at the University of Arizona.

## Supplementary Material

Additional file 1 **Supplementary Figures**. This file contains additional figures (SF1-SF9) not included in the main document. Click here for file

Additional file 2 **Supplementary Tables**. This file contains additional tables (ST1-ST7) not included in the main document. Click here for file
