# Supplementary material for: GC3 biology in corn, rice, sorghum and other grasses
Source: BMC Genomics. 2010 May 16;11:308. doi: 10.1186/1471-2164-11-308 (PMC2895627; doi:10.1186/1471-2164-11-308)
Supplement: Additional file 1 — Supplementary Figures. This file contains additional figures (SF1-SF9) not included in the main document. [file 1471-2164-11-308-S1.DOCX]

**Supplementary figures**


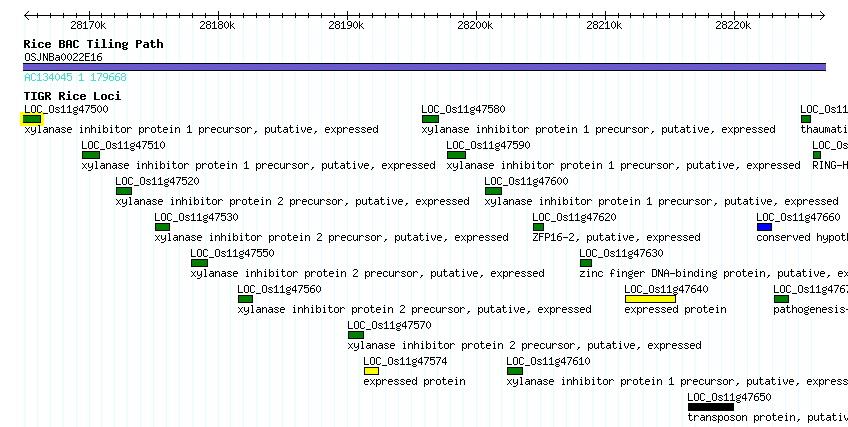


Supplementary Figure SF1: Cluster of high GC_3_ genes on rice chromosome 11, as shown in TIGR genome browser ([www.tigr.org](http://www.tigr.org))

Supplementary Figure SF2: Frequency of a canonical TATA-box in the 50 nucleotides upstream of the transcription start site for TSS predictions refined using the TSSer algorithm. Overall, 48% of rice promoters contain a canonical TATA-box; only 33% of genes with GC_3_ < 0.45 are equipped with TATA+ promoters as compared to 68% of those with GC_3_ > 0.95.

**Supplementary Figure SF3: Intron density as a function of GC_3_ in *Oryza sativa* and *Sorghum bicolor.* Intron density is defined as a number of introns per 1000 nucleotides of transcript length.**

**Supplementary Figure SF4: Distribution of dinucleotides CG and GC in *O. sativa*, stratified by high (CG_3_>0.8) and low (CG_3_<0.8) CG content in the third position of the codon.**

Supplementary Figure SF5: Relative abundances ρC_3_G_1_ and ρC_2_G_3_ of GC at the wobble position. For rice and sorghum, we defined GC_3_ high is >0.8 and GC_3_ low is <0.5. For arabidopsis, we assumed GC_3_ high >0.6 and GC_3_ low<0.3.

Supplementary Figure SF6: Distribution of position-specific relative abundance HEAT repeat, *O. sativa*.

Supplementary Figure SF7: Distribution of position-specific relative abundance for protein kinase families, *O. sativa*.

Supplementary Figure SF8: Distribution GC_3_ in honey bee (*Apis mellifera*)*,* fruit fly (*Drosophila melanogaster*)*,* and fire ant (*Solenopsis invicta*). Generally low GC_3_ organism honey bee has a surprising medium and high GC_3_ tail, containing approximately 25% of genes. This tail consists of a bunch of various “receptor” encoding genes (e.g. Metabotropic glutamate receptor, Toll-like receptor, Dopamine receptor type D2, D2-like dopamine receptor, Ephrin receptor, SIFamide receptor, Ecdysteroid receptor A isoform, Antennapedia protein, Nicotinic acetylcholine receptor alpha1 subunit, Alpha-glycosidase G-protein coupled receptor, etc). Based on current annotation, 2.2% of all *Apis mellifera* genes encode receptors. Considering two extremes (1000 genes with highest and lowest values of GC_3_), lowest GC_3_ genes encode receptors in 1.3% of cases as compared with 5.6% of high GC_3_ genes.

**Supplementary Figure SF9: Distribution GC_3_ in *Brachypodium distachyon.* Distribution is bimodal with high (~47%) fraction of ORFs having GC_3_ ≥0.8. Since this analysis was done based on available collection of *Brachypodium distachyon* ESTs (**[**www.brachypodium.org**](www.brachypodium.org)**), this effect may be due to sequence redundancy.**
