# Supplementary material for: GC3 biology in corn, rice, sorghum and other grasses
Source: BMC Genomics. 2010 May 16;11:308. doi: 10.1186/1471-2164-11-308 (PMC2895627; doi:10.1186/1471-2164-11-308)
Supplement: Additional file 2 — Supplementary Tables. This file contains additional tables (ST1-ST7) not included in the main document. [file 1471-2164-11-308-S2.DOCX]

Supplementary Table ST1: Clumps of high-GC_3_ genes in *O. sativa*

| CLUMP_ID | CLUMP_SIZE | LOCUS | GC3 | PFAM |
| --- | --- | --- | --- | --- |
| 1 | 13 | Os11g47500 | 0.914754 | Glyco_hydro_18 |
| 1 | 13 | Os11g47510 | 0.936102 | Glyco_hydro_18 |
| 1 | 13 | Os11g47520 | 0.931271 | Glyco_hydro_18 |
| 1 | 13 | Os11g47530 | 0.935154 | Glyco_hydro_18 |
| 1 | 13 | Os11g47550 | 0.931034 | Glyco_hydro_18 |
| 1 | 13 | Os11g47560 | 0.957895 | Glyco_hydro_18 |
| 1 | 13 | Os11g47574 | 0.461538 |  |
| 1 | 13 | Os11g47580 | 0.95082 | Glyco_hydro_18 |
| 1 | 13 | Os11g47590 | 0.940199 | Glyco_hydro_18 |
| 1 | 13 | Os11g47600 | 0.943709 | Glyco_hydro_18 |
| 1 | 13 | Os11g47610 | 0.930693 | Glyco_hydro_18 |
| 1 | 13 | Os11g47630 | 0.871345 | zf-C2H2 |
| 1 | 13 | Os11g47670 | 0.971831 | Thaumatin |
| 2 | 10 | Os11g05614 | 0.871186 | NAM |
| 2 | 10 | Os11g05660 | 0.952978 | F-box |
| 2 | 10 | Os11g05730 | 0.970803 | Histone |
| 2 | 10 | Os11g05740 | 0.846429 | B3 |
| 2 | 10 | Os11g05760 | 0.816406 | Peptidase_S28 |
| 2 | 10 | Os11g05770 | 0.850877 | DUF623 |
| 2 | 10 | Os11g05810 | 0.87766 |  |
| 2 | 10 | Os11g05850 | 0.530822 |  |
| 2 | 10 | Os11g05860 | 0.981043 | Hin1 |
| 2 | 10 | Os11g05870 | 0.860058 | Hin1 |
| 3 | 11 | Os10g40280 | 0.84507 |  |
| 3 | 11 | Os10g40360 | 0.936864 | Pro_dh |
| 3 | 11 | Os10g40370 | 0.596154 |  |
| 3 | 11 | Os10g40410 | 0.734177 |  |
| 3 | 11 | Os10g40420 | 0.91411 | Tryp_alpha_amyl |
| 3 | 11 | Os10g40430 | 0.938776 | Tryp_alpha_amyl |
| 3 | 11 | Os10g40440 | 0.881119 | Tryp_alpha_amyl |
| 3 | 11 | Os10g40490 | 0.509259 | U-box |
| 3 | 11 | Os10g40510 | 0.947761 | Tryp_alpha_amyl |
| 3 | 11 | Os10g40520 | 0.968 | Tryp_alpha_amyl |
| 3 | 11 | Os10g40530 | 0.962406 | Tryp_alpha_amyl |
| 4 | 10 | Os10g39000 | 0.925651 |  |
| 4 | 10 | Os10g39010 | 0.926627 | Pkinase_Tyr |
| 4 | 10 | Os10g39020 | 0.952652 | DUF604 |
| 4 | 10 | Os10g39030 | 0.901193 | Homeobox |
| 4 | 10 | Os10g39090 | 0.927273 |  |
| 4 | 10 | Os10g39120 | 0.596273 | UQ_con |
| 4 | 10 | Os10g39130 | 0.683761 | K-box |
| 4 | 10 | Os10g39140 | 0.915452 | 2OG-FeII_Oxy |
| 4 | 10 | Os10g39150 | 0.797688 |  |
| 4 | 10 | Os10g39170 | 0.970501 | peroxidase |
| 5 | 21 | Os10g38340 | 0.875 | GST_C, GTS_N |
| 5 | 21 | Os10g38360 | 0.905983 | GST_C, GTS_N |
| 5 | 21 | Os10g38450 | 0.691275 | LRRNT_2 |
| 5 | 21 | Os10g38470 | 0.95279 | GST_C, GTS_N |
| 5 | 21 | Os10g38495 | 0.866667 |  |
| 5 | 21 | Os10g38501 | 0.888889 |  |
| 5 | 21 | Os10g38540 | 0.862069 | GST_C, GTS_N |
| 5 | 21 | Os10g38580 | 0.901639 | GST_C, GTS_N |
| 5 | 21 | Os10g38590 | 0.908714 | GST_C, GTS_N |
| 5 | 21 | Os10g38610 | 0.902128 | GST_C, GTS_N |
| 5 | 21 | Os10g38670 | 0.894068 | GST_C, GTS_N |
| 5 | 21 | Os10g38700 | 0.917355 | GST_C, GTS_N |
| 5 | 21 | Os10g38710 | 0.931624 | GST_C, GTS_N |
| 5 | 21 | Os10g38720 | 0.909836 | GST_C, GTS_N |
| 5 | 21 | Os10g38740 | 0.940928 | GST_C, GTS_N |
| 5 | 21 | Os10g38780 | 0.949153 | GST_C, GTS_N |
| 5 | 21 | Os10g38820 | 0.852868 | bZIP_1 |
| 5 | 21 | Os10g38834 | 0.909357 | NAM |
| 5 | 21 | Os10g38860 | 0.888186 | Abhydrolase_1 |
| 5 | 21 | Os10g38870 | 0.955556 | HMA |
| 5 | 21 | Os10g38880 | 0.94697 | DUF623 |
| 6 | 10 | Os09g31300 | 0.90796 | HLH |
| 6 | 10 | Os09g31310 | 0.96988 | Acetyltransf_1 |
| 6 | 10 | Os09g31360 | 0.679089 |  |
| 6 | 10 | Os09g31390 | 0.830153 | bZIP_1 |
| 6 | 10 | Os09g31400 | 0.531046 | EIN3 |
| 6 | 10 | Os09g31430 | 0.706587 | Glyco_hydro_1 |
| 6 | 10 | Os09g31446 | 0.861878 |  |
| 6 | 10 | Os09g31454 | 0.899691 | Myb_DNA-binding |
| 6 | 10 | Os09g31458 | 0.883152 |  |
| 6 | 10 | Os09g31466 | 0.805085 |  |
| 7 | 14 | Os09g30490 | 0.934783 | efhand |
| 7 | 14 | Os09g30506 | 0.905797 | efhand |
| 7 | 14 | Os09g30510 | 0.819853 |  |
| 7 | 14 | Os09g31000 | 0.928058 | efhand |
| 7 | 14 | Os09g31031 | 0.80137 | Ribosomal_L40e |
| 7 | 14 | Os09g31040 | 0.826087 |  |
| 7 | 14 | Os09g31050 | 0.913889 |  |
| 7 | 14 | Os09g31080 | 0.940397 |  |
| 7 | 14 | Os09g31120 | 0.932886 | Pirin |
| 7 | 14 | Os09g31130 | 0.908088 | Na_sulph_symp |
| 7 | 14 | Os09g31170 | 0.843575 |  |
| 7 | 14 | Os09g31180 | 0.931937 | Ribosomal_L6 |
| 7 | 14 | Os09g31200 | 0.915152 | zf-AN1 |
| 7 | 14 | Os09g31210 | 0.95614 | Pkinase |
| 8 | 11 | Os09g29100 | 0.7507 | Cyclin_C |
| 8 | 11 | Os09g29120 | 0.937824 | GRAM |
| 8 | 11 | Os09g29130 | 0.925 | ZF-HD_dimer |
| 8 | 11 | Os09g29160 | 0.944196 |  |
| 8 | 11 | Os09g29170 | 0.654185 | Pkinase |
| 8 | 11 | Os09g29190 | 0.539604 | SAICAR_synt |
| 8 | 11 | Os09g29200 | 0.924107 | GST_C |
| 8 | 11 | Os09g29210 | 0.920716 | DUF6 |
| 8 | 11 | Os09g29239 | 0.933702 | DUF6 |
| 8 | 11 | Os09g29270 | 0.751479 |  |
| 8 | 11 | Os09g29284 | 0.780538 | MatE |
| 9 | 17 | Os09g28120 | 0.811189 | F-box |
| 9 | 17 | Os09g28150 | 0.767857 | Carb_anhydrase |
| 9 | 17 | Os09g28180 | 0.907675 | B_lectin |
| 9 | 17 | Os09g28210 | 0.898734 | HLH |
| 9 | 17 | Os09g28230 | 0.847352 | Abhydrolase_3 |
| 9 | 17 | Os09g28280 | 0.834375 | Abhydrolase_3 |
| 9 | 17 | Os09g28300 | 0.513688 | Remorin_C |
| 9 | 17 | Os09g28310 | 0.769231 | bZIP_1 |
| 9 | 17 | Os09g28340 | 0.881612 | SMI1_KNR4 |
| 9 | 17 | Os09g28354 | 0.90099 | HSF_DNA-bind |
| 9 | 17 | Os09g28400 | 0.907029 | Alpha-amylase |
| 9 | 17 | Os09g28430 | 0.796804 |  |
| 9 | 17 | Os09g28440 | 0.8 | AP2 |
| 9 | 17 | Os09g28460 | 0.891501 | XG_FTase |
| 9 | 17 | Os09g28470 | 0.798434 | LRRNT_2 |
| 9 | 17 | Os09g28489 | 0.788991 |  |
| 9 | 17 | Os09g28520 | 0.879747 |  |
| 10 | 10 | Os08g36860 | 0.900196 | p450 |
| 10 | 10 | Os08g36900 | 0.917808 | Alpha-amylase |
| 10 | 10 | Os08g36910 | 0.91195 | Alpha-amylase |
| 10 | 10 | Os08g36970 | 0.850806 |  |
| 10 | 10 | Os08g36980 | 0.394286 | zf-CSL |
| 10 | 10 | Os08g37040 | 0.967066 | Abhydrolase_3 |
| 10 | 10 | Os08g37060 | 0.972561 | Abhydrolase_3 |
| 10 | 10 | Os08g37130 | 0.94186 | adh_short |
| 10 | 10 | Os08g37180 | 0.941995 | Patatin |
| 10 | 10 | Os08g37210 | 0.93287 | Patatin |
| 11 | 13 | Os08g36150 | 0.843137 | AHSA1 |
| 11 | 13 | Os08g36170 | 0.875 | zf-C3HC4 |
| 11 | 13 | Os08g36250 | 0.73125 |  |
| 11 | 13 | Os08g36310 | 0.89022 | p450 |
| 11 | 13 | Os08g36320 | 0.537549 | Pyridoxal_deC |
| 11 | 13 | Os08g36340 | 0.853868 | K_trans |
| 11 | 13 | Os08g36390 | 0.859551 | zf-C2H2 |
| 11 | 13 | Os08g36420 | 0.523046 | Zip |
| 11 | 13 | Os08g36440 | 0.873134 | TB2_DP1_HVA22 |
| 11 | 13 | Os08g36480 | 0.966194 | Cyt-b5 |
| 11 | 13 | Os08g36540 | 0.914439 |  |
| 11 | 13 | Os08g36580 | 0.739837 |  |
| 11 | 13 | Os08g36630 | 0.858696 | Carb_anhydrase |
| 12 | 12 | Os08g34240 | 0.964602 | NAF |
| 12 | 12 | Os08g34270 | 0.951613 |  |
| 12 | 12 | Os08g34280 | 0.950276 | Epimerase |
| 12 | 12 | Os08g34290 | 0.635088 |  |
| 12 | 12 | Os08g34330 | 0.874644 | Str_synth |
| 12 | 12 | Os08g34340 | 0.44489 | DUF593 |
| 12 | 12 | Os08g34360 | 0.838095 | AP2 |
| 12 | 12 | Os08g34380 | 0.905744 | LRRNT_2 |
| 12 | 12 | Os08g34390 | 0.653509 |  |
| 12 | 12 | Os08g34460 | 0.97482 | Tetraspannin |
| 12 | 12 | Os08g34550 | 0.926295 | zf-C3HC4 |
| 12 | 12 | Os08g34580 | 0.837576 | Glyco_transf_20 |
| 13 | 10 | Os08g01940 | 0.791173 | DUF608 |
| 13 | 10 | Os08g01950 | 0.9375 | Transferase |
| 13 | 10 | Os08g02030 | 0.884861 | Transferase |
| 13 | 10 | Os08g02040 | 0.585329 | PPR |
| 13 | 10 | Os08g02060 | 0.72973 |  |
| 13 | 10 | Os08g02070 | 0.668161 | K-box |
| 13 | 10 | Os08g02080 | 0.916084 | Rick_17kDa_Anti |
| 13 | 10 | Os08g02094 | 0.934555 | Lipase_GDSL |
| 13 | 10 | Os08g02110 | 0.977918 | peroxidase |
| 13 | 10 | Os08g02120 | 0.95549 | PfkB |
| 14 | 10 | Os06g38990 | 0.893756 | LRRNT_2 |
| 14 | 10 | Os06g39050 | 0.97411 | SNARE |
| 14 | 10 | Os06g39060 | 0.966942 | Glyco_hydro_17 |
| 14 | 10 | Os06g39110 | 0.692913 |  |
| 14 | 10 | Os06g39120 | 0.632 |  |
| 14 | 10 | Os06g39140 | 0.705202 | Bac_globin |
| 14 | 10 | Os06g39240 | 0.980769 | HTH_3 |
| 14 | 10 | Os06g39260 | 0.679666 | UAA |
| 14 | 10 | Os06g39270 | 0.94332 | UDPGT |
| 14 | 10 | Os06g39330 | 0.951064 | UDPGT |
| 15 | 12 | Os06g34730 | 0.889423 |  |
| 15 | 12 | Os06g34790 | 0.993976 | DUF538 |
| 15 | 12 | Os06g34830 | 0.979899 | AA_permease |
| 15 | 12 | Os06g34850 | 0.850746 |  |
| 15 | 12 | Os06g35030 | 0.636364 | RRM_1 |
| 15 | 12 | Os06g35050 | 0.781341 | PDH |
| 15 | 12 | Os06g35140 | 0.78125 | Myb_DNA-binding |
| 15 | 12 | Os06g35160 | 0.95534 | NAF |
| 15 | 12 | Os06g35320 | 0.910412 | Glyco_hydro_28 |
| 15 | 12 | Os06g35410 | 0.801325 | DUF246 |
| 15 | 12 | Os06g35480 | 0.962382 | peroxidase |
| 15 | 12 | Os06g35520 | 0.909341 | peroxidase |
| 16 | 13 | Os06g10790 | 0.938115 | Lectin_legB |
| 16 | 13 | Os06g10850 | 0.906122 | Lipase_3 |
| 16 | 13 | Os06g10880 | 0.836923 | bZIP_1 |
| 16 | 13 | Os06g10910 | 0.941696 | XG_FTase |
| 16 | 13 | Os06g10930 | 0.926391 | XG_FTase |
| 16 | 13 | Os06g10970 | 0.902827 | XG_FTase |
| 16 | 13 | Os06g10990 | 0.779193 | HSP70 |
| 16 | 13 | Os06g11020 | 0.7897 | Tic22 |
| 16 | 13 | Os06g11040 | 0.512315 | DUF829 |
| 16 | 13 | Os06g11060 | 0.939655 |  |
| 16 | 13 | Os06g11070 | 0.553892 |  |
| 16 | 13 | Os06g11090 | 0.962848 | Abhydrolase_3 |
| 16 | 13 | Os06g11130 | 0.963636 | Abhydrolase_3 |
| 17 | 15 | Os06g02630 | 0.8 |  |
| 17 | 15 | Os06g02730 | 0.920455 |  |
| 17 | 15 | Os06g02780 | 0.880808 |  |
| 17 | 15 | Os06g02830 | 0.854701 | DUF594 |
| 17 | 15 | Os06g02850 | 0.955671 | DUF594 |
| 17 | 15 | Os06g02900 | 0.858871 |  |
| 17 | 15 | Os06g02940 | 0.821918 |  |
| 17 | 15 | Os06g02960 | 0.917862 | DUF594 |
| 17 | 15 | Os06g03080 | 0.909707 |  |
| 17 | 15 | Os06g03099 | 0.92511 |  |
| 17 | 15 | Os06g03150 | 0.925743 | DUF594 |
| 17 | 15 | Os06g03220 | 0.718644 |  |
| 17 | 15 | Os06g03390 | 0.640625 |  |
| 17 | 15 | Os06g03486 | 0.813559 |  |
| 17 | 15 | Os06g03520 | 0.917241 | DUF581 |
| 18 | 10 | Os05g41950 | 0.804124 | Pkinase |
| 18 | 10 | Os05g41990 | 0.963303 | peroxidase |
| 18 | 10 | Os05g42000 | 0.94 | peroxidase |
| 18 | 10 | Os05g42020 | 0.831622 | UDPGT |
| 18 | 10 | Os05g42040 | 0.77732 | UDPGT |
| 18 | 10 | Os05g42100 | 0.906158 | DUF1995 |
| 18 | 10 | Os05g42120 | 0.941176 |  |
| 18 | 10 | Os05g42130 | 0.946009 | GRAS |
| 18 | 10 | Os05g42150 | 0.97619 | GH3 |
| 18 | 10 | Os05g42190 | 0.88835 | Flavodoxin_1 |
| 19 | 12 | Os05g35240 | 0.890052 |  |
| 19 | 12 | Os05g35260 | 0.945504 | PB1 |
| 19 | 12 | Os05g35266 | 0.951456 | Galactosyl_T |
| 19 | 12 | Os05g35340 | 0.447059 |  |
| 19 | 12 | Os05g35400 | 0.969477 | HSP70 |
| 19 | 12 | Os05g35410 | 0.884943 | Ank |
| 19 | 12 | Os05g35440 | 0.690476 | SHMT |
| 19 | 12 | Os05g35444 | 0.756757 |  |
| 19 | 12 | Os05g35460 | 0.854949 | CRAL_TRIO |
| 19 | 12 | Os05g35470 | 0.948718 | DLH |
| 19 | 12 | Os05g35480 | 0.757764 | C2 |
| 19 | 12 | Os05g35500 | 0.923372 | Myb_DNA-binding |
| 20 | 16 | Os04g48830 | 0.834239 | DUF623 |
| 20 | 16 | Os04g48850 | 0.915289 | Aminotran_1_2 |
| 20 | 16 | Os04g48860 | 0.726667 |  |
| 20 | 16 | Os04g48880 | 0.903226 | FA_hydroxylase |
| 20 | 16 | Os04g48930 | 0.809886 | FAD_binding_8 |
| 20 | 16 | Os04g48950 | 0.902857 | DUF604 |
| 20 | 16 | Os04g49000 | 0.953052 | zf-C3HC4 |
| 20 | 16 | Os04g49060 | 0.754591 |  |
| 20 | 16 | Os04g49110 | 0.856452 | GRAS |
| 20 | 16 | Os04g49140 | 0.742604 | DUF1191 |
| 20 | 16 | Os04g49150 | 0.596078 | K-box |
| 20 | 16 | Os04g49160 | 0.920245 | zf-C3HC4 |
| 20 | 16 | Os04g49194 | 0.83871 | 2OG-FeII_Oxy |
| 20 | 16 | Os04g49210 | 0.883853 | 2OG-FeII_Oxy |
| 20 | 16 | Os04g49250 | 0.748691 | UDPG_MGDP_dh |
| 20 | 16 | Os04g49260 | 0.829268 | HMA |
| 21 | 19 | Os04g46400 | 0.898246 | AP2 |
| 21 | 19 | Os04g46440 | 0.93578 | AP2 |
| 21 | 19 | Os04g46444 | 0.697917 |  |
| 21 | 19 | Os04g46460 | 0.834302 | AA_kinase |
| 21 | 19 | Os04g46470 | 0.870492 | RPE65 |
| 21 | 19 | Os04g46480 | 0.597855 |  |
| 21 | 19 | Os04g46490 | 0.940741 | MIP |
| 21 | 19 | Os04g46560 | 0.813031 | Ldh_1_C |
| 21 | 19 | Os04g46570 | 0.478 | DUF246 |
| 21 | 19 | Os04g46580 | 0.844875 | SBP |
| 21 | 19 | Os04g46600 | 0.928 |  |
| 21 | 19 | Os04g46610 | 0.747748 | PAP_fibrillin |
| 21 | 19 | Os04g46620 | 0.430403 | Cpn60_TCP1 |
| 21 | 19 | Os04g46650 | 0.891304 | DPBB_1 |
| 21 | 19 | Os04g46660 | 0.958716 | Cyclin |
| 21 | 19 | Os04g46670 | 0.739054 | zf-C2H2 |
| 21 | 19 | Os04g46680 | 0.833333 | zf-C2H2 |
| 21 | 19 | Os04g46700 | 0.75942 | DUF140 |
| 21 | 19 | Os04g46740 | 0.932203 | Pectinesterase |
| 22 | 11 | Os04g44240 | 0.874751 | UDPGT |
| 22 | 11 | Os04g44280 | 0.866667 | Response_reg |
| 22 | 11 | Os04g44290 | 0.605 |  |
| 22 | 11 | Os04g44340 | 0.718631 | DUF177 |
| 22 | 11 | Os04g44354 | 0.910448 | UDPGT |
| 22 | 11 | Os04g44400 | 0.874194 | CLP_protease |
| 22 | 11 | Os04g44410 | 0.94198 | Peptidase_S10 |
| 22 | 11 | Os04g44440 | 0.932367 | TCP |
| 22 | 11 | Os04g44470 | 0.935323 | Kunitz_legume |
| 22 | 11 | Os04g44500 | 0.901709 | GRAM |
| 22 | 11 | Os04g44510 | 0.842593 | GRAM |
| 23 | 20 | Os04g33360 | 0.916667 | 2OG-FeII_Oxy |
| 23 | 20 | Os04g33370 | 0.986767 | p450 |
| 23 | 20 | Os04g33390 | 0.956522 | PDT |
| 23 | 20 | Os04g33450 | 0.80117 | DUF662 |
| 23 | 20 | Os04g33470 | 0.697531 | Metallophos |
| 23 | 20 | Os04g33510 | 0.416667 |  |
| 23 | 20 | Os04g33520 | 0.969101 | Root_cap |
| 23 | 20 | Os04g33530 | 0.900662 | Metallophos |
| 23 | 20 | Os04g33570 | 0.827586 | PBP |
| 23 | 20 | Os04g33590 | 0.854396 | Abhydrolase_1 |
| 23 | 20 | Os04g33610 | 0.853211 |  |
| 23 | 20 | Os04g33630 | 0.908497 | Fer2 |
| 23 | 20 | Os04g33640 | 0.908629 | Glyco_hydro_17 |
| 23 | 20 | Os04g33660 | 0.727599 | Carb_anhydrase |
| 23 | 20 | Os04g33670 | 0.828244 |  |
| 23 | 20 | Os04g33680 | 0.922049 | Glutaredoxin |
| 23 | 20 | Os04g33690 | 0.482432 | Sec23_BS |
| 23 | 20 | Os04g33710 | 0.868932 |  |
| 23 | 20 | Os04g33720 | 0.954003 | Glyco_hydro_32C |
| 23 | 20 | Os04g33740 | 0.906511 | Glyco_hydro_32C |
| 24 | 16 | Os04g01500 | 0.938931 |  |
| 24 | 16 | Os04g01510 | 0.90303 | DUF1295 |
| 24 | 16 | Os04g01520 | 0.798982 | DUF895 |
| 24 | 16 | Os04g01540 | 0.761905 |  |
| 24 | 16 | Os04g01570 | 0.962617 | PMEI |
| 24 | 16 | Os04g01590 | 0.530792 | Arginase |
| 24 | 16 | Os04g01600 | 0.927273 | 2-Hacid_dh |
| 24 | 16 | Os04g01660 | 0.941896 | 2-Hacid_dh |
| 24 | 16 | Os04g01674 | 0.921136 | 2-Hacid_dh |
| 24 | 16 | Os04g01690 | 0.935897 | Orn_Arg_deC_N |
| 24 | 16 | Os04g01710 | 0.778646 | Inhibitor_I29 |
| 24 | 16 | Os04g01740 | 0.963068 | HATPase_c |
| 24 | 16 | Os04g01874 | 0.818306 | Lectin_legB |
| 24 | 16 | Os04g01910 | 0.889908 | Lectin_legB |
| 24 | 16 | Os04g01950 | 0.86901 | Lectin_legB |
| 24 | 16 | Os04g01960 | 0.994975 |  |
| 25 | 10 | Os03g22170 | 0.918033 | AP2 |
| 25 | 10 | Os03g22180 | 0.824468 | Ribosomal_L18e |
| 25 | 10 | Os03g22210 | 0.934426 |  |
| 25 | 10 | Os03g22230 | 0.947977 |  |
| 25 | 10 | Os03g22259 | 0.756098 |  |
| 25 | 10 | Os03g22270 | 0.843284 | Auxin_repressed |
| 25 | 10 | Os03g22330 | 0.767391 | DNA_photolyase |
| 25 | 10 | Os03g22340 | 0.94697 | Ribosomal_L22e |
| 25 | 10 | Os03g22350 | 0.631579 | Brix |
| 25 | 10 | Os03g22370 | 0.922481 | PsbX |
| 26 | 13 | Os03g14010 | 0.888298 | Glyco_hydro_10 |
| 26 | 13 | Os03g14030 | 0.916914 | Thaumatin |
| 26 | 13 | Os03g14040 | 0.683871 |  |
| 26 | 13 | Os03g14050 | 0.888889 | Thaumatin |
| 26 | 13 | Os03g14080 | 0.92381 | Aa_trans |
| 26 | 13 | Os03g14090 | 0.891808 | Arm |
| 26 | 13 | Os03g14120 | 0.42638 | DapB_N |
| 26 | 13 | Os03g14130 | 0.857143 | DUF1210 |
| 26 | 13 | Os03g14140 | 0.837302 | DUF1210 |
| 26 | 13 | Os03g14150 | 0.91746 |  |
| 26 | 13 | Os03g14170 | 0.977486 | ACP_syn_III_C |
| 26 | 13 | Os03g14180 | 0.966805 | HSP20 |
| 26 | 13 | Os03g14210 | 0.893271 | Glyco_hydro_17 |
| 27 | 11 | Os03g08790 | 0.972665 | Asp |
| 27 | 11 | Os03g08810 | 0.686695 |  |
| 27 | 11 | Os03g08840 | 0.875622 |  |
| 27 | 11 | Os03g08850 | 0.80298 |  |
| 27 | 11 | Os03g08860 | 0.618868 | GRAM |
| 27 | 11 | Os03g08880 | 0.9375 | TPT |
| 27 | 11 | Os03g08920 | 0.90678 | zf-C3HC4 |
| 27 | 11 | Os03g08930 | 0.863636 | HLH |
| 27 | 11 | Os03g08960 | 0.897436 | HALZ |
| 27 | 11 | Os03g08970 | 0.858757 |  |
| 27 | 11 | Os03g08980 | 0.89749 |  |
| 28 | 13 | Os02g43030 | 0.918455 | DUF125 |
| 28 | 13 | Os02g43120 | 0.974747 | zf-C3HC4 |
| 28 | 13 | Os02g43130 | 0.477966 | Kinesin |
| 28 | 13 | Os02g43150 | 0.930556 | GATA |
| 28 | 13 | Os02g43170 | 0.925926 | zf-B_box |
| 28 | 13 | Os02g43180 | 0.515152 | Glutaredoxin |
| 28 | 13 | Os02g43194 | 0.754098 | Aldedh |
| 28 | 13 | Os02g43250 | 0.904762 | LRR_1 |
| 28 | 13 | Os02g43280 | 0.707317 | Aldedh |
| 28 | 13 | Os02g43290 | 0.939891 | Pkinase |
| 28 | 13 | Os02g43300 | 0.893482 |  |
| 28 | 13 | Os02g43314 | 0.65942 |  |
| 28 | 13 | Os02g43330 | 0.919847 | HALZ |
| 29 | 10 | Os02g41630 | 0.964387 | PAL |
| 29 | 10 | Os02g41650 | 0.920723 | PAL |
| 29 | 10 | Os02g41680 | 0.864146 | PAL |
| 29 | 10 | Os02g41780 | 0.675439 | TPT |
| 29 | 10 | Os02g41840 | 0.918129 | DUF584 |
| 29 | 10 | Os02g41860 | 0.934483 | MIP |
| 29 | 10 | Os02g41890 | 0.475783 | LRRNT_2 |
| 29 | 10 | Os02g41904 | 0.950617 | Gamma-thionin |
| 29 | 10 | Os02g41910 | 0.846535 | F-box |
| 29 | 10 | Os02g41954 | 0.944444 | 2OG-FeII_Oxy |
| 30 | 14 | Os01g72360 | 0.949772 |  |
| 30 | 14 | Os01g72370 | 0.779116 | HLH |
| 30 | 14 | Os01g72380 | 0.948953 | DUF594 |
| 30 | 14 | Os01g72410 | 0.575707 | NB-ARC |
| 30 | 14 | Os01g72420 | 0.929648 | C2 |
| 30 | 14 | Os01g72430 | 0.661616 | FMN_red |
| 30 | 14 | Os01g72460 | 0.740196 | FMN_red |
| 30 | 14 | Os01g72470 | 0.784091 |  |
| 30 | 14 | Os01g72480 | 0.590747 | zf-C3HC4 |
| 30 | 14 | Os01g72490 | 0.88563 | DUF702 |
| 30 | 14 | Os01g72510 | 0.903587 | Asp |
| 30 | 14 | Os01g72520 | 0.894434 | Phosphoesterase |
| 30 | 14 | Os01g72530 | 0.921053 | efhand |
| 30 | 14 | Os01g72610 | 0.947154 | DUF563 |
| 31 | 15 | Os01g64100 | 0.95302 | Glyco_hydro_18 |
| 31 | 15 | Os01g64110 | 0.934641 | Glyco_hydro_18 |
| 31 | 15 | Os01g64120 | 0.885542 | Fer2 |
| 31 | 15 | Os01g64170 | 0.922306 | Glyco_hydro_17 |
| 31 | 15 | Os01g64190 | 0.852713 |  |
| 31 | 15 | Os01g64262 | 0.812834 | Abhydrolase_1 |
| 31 | 15 | Os01g64270 | 0.828877 |  |
| 31 | 15 | Os01g64280 | 0.4275 | PX |
| 31 | 15 | Os01g64290 | 0.949721 |  |
| 31 | 15 | Os01g64300 | 0.786364 | DUF584 |
| 31 | 15 | Os01g64310 | 0.832685 | NAM |
| 31 | 15 | Os01g64370 | 0.471572 |  |
| 31 | 15 | Os01g64430 | 0.960699 | DUF623 |
| 31 | 15 | Os01g64450 | 0.950413 | Hin1 |
| 31 | 15 | Os01g64470 | 0.912134 | Hin1 |
| 32 | 11 | Os01g63460 | 0.893333 | Myb_DNA-binding |
| 32 | 11 | Os01g63470 | 0.719512 | C2 |
| 32 | 11 | Os01g63480 | 0.896694 | Transferase |
| 32 | 11 | Os01g63500 | 0.827338 |  |
| 32 | 11 | Os01g63510 | 0.870647 | Homeobox |
| 32 | 11 | Os01g63540 | 0.906593 | p450 |
| 32 | 11 | Os01g63620 | 0.883041 |  |
| 32 | 11 | Os01g63690 | 0.909474 | Hs1pro-1_C |
| 32 | 11 | Os01g63710 | 0.517949 | Cdc6_C |
| 32 | 11 | Os01g63770 | 0.955375 | Aa_trans |
| 32 | 11 | Os01g63800 | 0.955975 | PH |
| 33 | 13 | Os01g53330 | 0.858333 | UDPGT |
| 33 | 13 | Os01g53350 | 0.903967 | UDPGT |
| 33 | 13 | Os01g53370 | 0.772358 | UDPGT |
| 33 | 13 | Os01g53390 | 0.767442 | UDPGT |
| 33 | 13 | Os01g53420 | 0.845703 | UDPGT |
| 33 | 13 | Os01g53430 | 0.867368 | UDPGT |
| 33 | 13 | Os01g53450 | 0.461197 | Aminotran_1_2 |
| 33 | 13 | Os01g53460 | 0.894068 | UDPGT |
| 33 | 13 | Os01g53470 | 0.988095 | Hin1 |
| 33 | 13 | Os01g53500 | 0.969697 | zf-C3HC4 |
| 33 | 13 | Os01g53520 | 0.700787 | Reticulon |
| 33 | 13 | Os01g53550 | 0.828452 |  |
| 33 | 13 | Os01g53570 | 0.839228 | UPF0005 |
| 34 | 16 | Os01g41120 | 0.865149 | LRR_1 |
| 34 | 16 | Os01g41140 | 0.766839 |  |
| 34 | 16 | Os01g41170 | 0.837945 |  |
| 34 | 16 | Os01g41220 | 0.968944 | DUF538 |
| 34 | 16 | Os01g41240 | 0.940594 | Abhydrolase_1 |
| 34 | 16 | Os01g41270 | 0.840074 | F-box |
| 34 | 16 | Os01g41290 | 0.817658 | F-box |
| 34 | 16 | Os01g41310 | 0.732632 | F-box |
| 34 | 16 | Os01g41370 | 0.762712 | FBD |
| 34 | 16 | Os01g41430 | 0.899598 | UDPGT |
| 34 | 16 | Os01g41510 | 0.388128 | efhand |
| 34 | 16 | Os01g41530 | 0.884393 | F-box |
| 34 | 16 | Os01g41550 | 0.966068 | Asp |
| 34 | 16 | Os01g41610 | 0.739837 | ATP-synt_G |
| 34 | 16 | Os01g41640 | 0.706667 |  |
| 34 | 16 | Os01g41710 | 0.996183 | Chloroa_b-bind |
| 35 | 16 | Os01g11430 | 0.763006 |  |
| 35 | 16 | Os01g11460 | 0.930108 | zf-C3HC4 |
| 35 | 16 | Os01g11520 | 0.844538 | zf-C3HC4 |
| 35 | 16 | Os01g11550 | 0.875817 | TCP |
| 35 | 16 | Os01g11570 | 0.677824 | Lipase_GDSL |
| 35 | 16 | Os01g11580 | 0.544944 | Bromodomain |
| 35 | 16 | Os01g11620 | 0.871935 | Lipase_GDSL |
| 35 | 16 | Os01g11650 | 0.949062 | Lipase_GDSL |
| 35 | 16 | Os01g11660 | 0.758904 | Lipase_GDSL |
| 35 | 16 | Os01g11700 | 0.864721 | Lipase_GDSL |
| 35 | 16 | Os01g11710 | 0.944591 | Lipase_GDSL |
| 35 | 16 | Os01g11730 | 0.874372 | Lipase_GDSL |
| 35 | 16 | Os01g11740 | 0.847545 | Lipase_GDSL |
| 35 | 16 | Os01g11810 | 0.936869 | DUF231 |
| 35 | 16 | Os01g11820 | 0.777174 |  |
| 35 | 16 | Os01g11860 | 0.813131 | DJ-1_PfpI |
| 36 | 27 | Os01g03530 | 0.941681 | Cu-oxidase |
| 36 | 27 | Os01g03549 | 0.961603 | Cu-oxidase_2 |
| 36 | 27 | Os01g03570 | 0.565062 | XH |
| 36 | 27 | Os01g03620 | 0.939597 | Cu-oxidase |
| 36 | 27 | Os01g03630 | 0.943925 | Cu-oxidase |
| 36 | 27 | Os01g03650 | 0.689441 | UPF0051 |
| 36 | 27 | Os01g03670 | 0.946746 | Epimerase |
| 36 | 27 | Os01g03680 | 0.933333 | Bowman-Birk_leg |
| 36 | 27 | Os01g03690 | 0.895604 |  |
| 36 | 27 | Os01g03710 | 0.601399 | PMI_typeI |
| 36 | 27 | Os01g03720 | 0.871795 | Myb_DNA-binding |
| 36 | 27 | Os01g03730 | 0.711974 | S1-P1_nuclease |
| 36 | 27 | Os01g03740 | 0.688356 | S1-P1_nuclease |
| 36 | 27 | Os01g03760 | 0.626917 |  |
| 36 | 27 | Os01g03810 | 0.630631 |  |
| 36 | 27 | Os01g03870 | 0.809148 | DUF1666 |
| 36 | 27 | Os01g03900 | 0.867347 |  |
| 36 | 27 | Os01g03914 | 0.737245 | Cation_efflux |
| 36 | 27 | Os01g03940 | 0.96875 |  |
| 36 | 27 | Os01g03950 | 0.900322 | Glyco_hydro_31 |
| 36 | 27 | Os01g03980 | 0.925714 |  |
| 36 | 27 | Os01g04020 | 0.728614 | AP2 |
| 36 | 27 | Os01g04050 | 0.80198 | Bowman-Birk_leg |
| 36 | 27 | Os01g04080 | 0.830579 |  |
| 36 | 27 | Os01g04090 | 0.7475 |  |
| 36 | 27 | Os01g04130 | 0.630769 | Pept_tRNA_hydro |
| 36 | 27 | Os01g04170 | 0.933333 |  |

Table ST2: Genome ontology and GC_3_. Genome of *A. thaliana* is AT-rich; its genes are well-characterized and have a tight GC_3_ distribution. Therefore, it can serve as a reference to compare GC_3_ for multiple organisms and classes of genes. We compared *Arabidopsis* genes with their orthologs (defined as best bi-directional hits) in the following GC_3_ rich organisms: *D. rerio, M. musculus, H. sapiens, C. reinhardtii, O. sativa* and *Z. mays*. *Arabidopsis* genes were grouped by their Gene Ontology annotation; unknown biological processes were excluded from this analysis. We used GC_3_ levels as a base line and computed absolute increase in GC3 as a difference between GC_3_ in an organism and GC_3_ of the corresponding ortholog in *A. thaliana*. If at least 4 organisms had an ortholog of an *Arabidopsis* gene, we averaged the increase in GC3 across all organisms. Then we computed average absolute GC_3_ increase within GO categories. The most significant results of increase (p-value <10^-100^) are presented in the Supplemental Table ST2. It appears that some classes of genes are under pressure in some genomes to become GC_3_ rich.

| GO TERM | ABSOLUTE GC3 INCREASE | STANDARD DEVIATION | NUMBER of GENES | NUMBER of ORGANISMS |
| --- | --- | --- | --- | --- |
| Electron transport or energy pathways | 0.253 | 0.182 | 560 | 4 |
| Response to abiotic or biotic stimulus | 0.245 | 0.173 | 1524 | 4 |
| Response to stress | 0.243 | 0.17 | 1450 | 4 |
| Transcription | 0.237 | 0.168 | 987 | 4 |
| Signal transduction | 0.212 | 0.167 | 626 | 4 |
| Transport | 0.209 | 0.164 | 1523 | 4 |
| Developmental processes | 0.196 | 0.163 | 1415 | 4 |
| Protein metabolism | 0.195 | 0.156 | 2877 | 4 |
| Cell organization and biogenesis | 0.193 | 0.159 | 1166 | 4 |
| DNA or RNA metabolism | 0.147 | 0.141 | 448 | 4 |

We have divided the genes of *O. sativa* into two groups by GC_3_ content: high group (GC_3_>0.8) with 4,889 members and low group (GC_3_<0.8) with 11,608 members. Genes annotated as “expressed proteins” are more prevalent in the low group (22% vs. 33%). Alpha-expansins are divided between high and low classes as 20:1 and are clearly more prevalent in the high group (relative abundance is 47.49); Histones are split 25:25, but because the low group is more than twice smaller, relative abundance of histones is 2.37; Ribosomal proteins are split 72:156, proportionally between high and low group. Absolute and relative abundance of 12 classes of *O. sativa* genes are shown in Supplemental Table ST3 below.

Supplemental Table ST3: Absolute and relative abundance of various classes of genes of *Oryza sativa* for high- and low- GC_3_ groups.

|  | Alpha-expansin | ACO | Embryo genesis | Anther specific | Chlorophyll | ids4 | Acyltransferase | Histone | Zinc finger | Heat shock | Trans. Fact. | Ribosomal |
| --- | --- | --- | --- | --- | --- | --- | --- | --- | --- | --- | --- | --- |
| High (count) | 20 | 11 | 11 | 23 | 10 | 2 | 20 | 25 | 111 | 33 | 72 | 72 |
| Low (count) | 1 | 1 | 2 | 8 | 5 | 1 | 17 | 25 | 149 | 52 | 119 | 156 |
| High (%) | 0.41 | 0.22 | 0.22 | 0.47 | 0.20 | 0.04 | 0.41 | 0.51 | 2.27 | 0.67 | 1.47 | 1.47 |
| Low (%) | 0.01 | 0.01 | 0.02 | 0.07 | 0.04 | 0.01 | 0.15 | 0.22 | 1.28 | 0.45 | 1.03 | 1.34 |
| High/ Low enrich-ment | 47.49 | 26.12 | 13.06 | 6.83 | 4.75 | 4.75 | 2.79 | 2.37 | 1.77 | 1.51 | 1.44 | 1.10 |

Table ST4: Conservation of relative abundance for GC and CWG between rice and sorghum, GO function.

| FUNCTION | CORRELATION of GC | CORRELATION of CWG | NUMBER OF PAIRS |
| --- | --- | --- | --- |
| motor activity | 0.367809 | 0.583312 | 19 |
| structural molecule activity | 0.795498 | 0.463891 | 54 |
| receptor activity | 0.836799 | 0.524704 | 77 |
| signal transducer activity | 0.866154 | 0.404981 | 61 |
| nucleic acid binding | 0.868789 | 0.644951 | 137 |
| binding | 0.869078 | 0.486697 | 322 |
| catalytic activity | 0.869934 | 0.481645 | 446 |
| kinase activity | 0.878198 | 0.470611 | 388 |
| enzyme regulator activity | 0.87996 | 0.393612 | 26 |
| transferase activity | 0.881816 | 0.416575 | 416 |
| hydrolase activity | 0.88538 | 0.463655 | 543 |
| transporter activity | 0.885523 | 0.408874 | 288 |
| protein binding | 0.892399 | 0.411737 | 449 |
| transcription regulator activity | 0.910993 | 0.429147 | 90 |

Table ST5: Conservation of relative abundance for GC and CWG between rice and sorghum, GO process.

| PROCESS | CORRELATION of GC PROFILES | CORRELATION of CWG PROFILES | NUMBER OF PAIRS COMPARED |
| --- | --- | --- | --- |
| catabolism | 0.763058 | 0.494327 | 88 |
| cell death | 0.776359 | 0.481317 | 29 |
| nucleobase, nucleoside, nucleotide and nucleic acid metabolism | 0.825419 | 0.548024 | 88 |
| amino acid and derivative metabolism | 0.836549 | 0.417623 | 156 |
| cell communication | 0.838131 | 0.722588 | 15 |
| metabolism | 0.842986 | 0.370685 | 133 |
| biosynthesis | 0.859286 | 0.456069 | 262 |
| development | 0.860211 | 0.503721 | 104 |
| transport | 0.89188 | 0.36693 | 230 |
| cell differentiation | 0.920557 | 0.610274 | 67 |

Table ST6: Conservation of relative abundance for GC and CWG between rice and Arabidopsis, GO function

| FUNCTION | CORRELATION of GC | CORRELATION of CWG | NUMBER OF PAIRS |
| --- | --- | --- | --- |
| motor activity | 0.126616 | 0.222371 | 123 |
| signal transducer activity | 0.254711 | 0.017356 | 412 |
| receptor activity | 0.325737 | 0.269151 | 332 |
| kinase activity | 0.380613 | 0.144903 | 1974 |
| protein binding | 0.382908 | 0.166266 | 2513 |
| transferase activity | 0.420016 | 0.144468 | 2175 |
| Binding | 0.42715 | 0.23955 | 1743 |
| catalytic activity | 0.430388 | 0.226112 | 2756 |
| structural molecule activity | 0.449682 | 0.154563 | 383 |
| transcription regulator activity | 0.466305 | 0.261971 | 515 |
| transporter activity | 0.467726 | 0.301953 | 1292 |

Table ST7: Conservation of relative abundance for GC and CWG between rice and Arabidopsis, GO process.

| PROCESS | CORRELATION of GC | CORRELATION of CWG | NUMBER OF PAIRS |
| --- | --- | --- | --- |
| cell communication | 0.091414 | 0.023371 | 101 |
| cell differentiation | 0.294193 | 0.228202 | 270 |
| cell death | 0.348146 | -0.04397 | 142 |
| biosynthesis | 0.406726 | 0.222069 | 1444 |
| catabolism | 0.425008 | 0.084355 | 623 |
| amino acid and derivative metabolism | 0.425826 | 0.183178 | 936 |
| transport | 0.457012 | 0.224103 | 1221 |
| cellular process | 0.483324 | 0.202604 | 2196 |
| metabolism | 0.489997 | 0.167435 | 766 |
| development | 0.517211 | 0.226717 | 571 |
| nucleobase, nucleoside, nucleotide and nucleic acid metabolism | 0.53221 | 0.382343 | 473 |
